# Dapagliflozin Preserves Peripheral Nerve Structure and Reduces Neuropathic Damage in Streptozotocin-Induced Diabetic Peripheral Neuropathy

**DOI:** 10.3390/ijms262412034

**Published:** 2025-12-14

**Authors:** Anca-Maria Țucă, Alexandra Nicoleta Preda, Georgică Târtea, Diana-Ruxandra Hădăreanu, Eugen Țieranu, Alexandra Oltea Dan, Elena-Anca Târtea, Andrei Greșiță, Denisa Floriana Vasilica Pîrșcoveanu, Veronica Sfredel, Smaranda Ioana Mitran

**Affiliations:** 1 Experimental Research Centre for Normal and Pathological Aging, University of Medicine and Pharmacy of Craiova, 2 Petru Rares St., 200349 Craiova, Romania; anca.tuca@umfcv.ro (A.-M.Ț.); alexandra.dan@umfcv.ro (A.O.D.); andrei.gresita@umfcv.ro (A.G.); veronica.sfredel@umfcv.ro (V.S.); smaranda.mitran@umfcv.ro (S.I.M.); 2 Department of Neurology, University of Medicine and Pharmacy of Craiova, 2 Petru Rares St., 200349 Craiova, Romania; anca.tartea@umfcv.ro (E.-A.T.); denisa.pirscoveanu@umfcv.ro (D.F.V.P.); 3 Department of Cardiology, University of Medicine and Pharmacy of Craiova, 2 Petru Rares St., 200349 Craiova, Romania; diana.hadareanu@umfcv.ro (D.-R.H.); eugen.tieranu@umfcv.ro (E.Ț.)

**Keywords:** dapagliflozin, diabetes mellitus, diabetic peripheral neuropathy, oxidative stress, neuroprotection

## Abstract

Diabetic peripheral neuropathy (DPN) is one of the most common chronic complications of diabetes mellitus, driven by oxidative stress, inflammation, and microvascular dysfunction. Dapagliflozin, a selective inhibitor of sodium–glucose cotransporter type 2 (SGLT2), is used in the treatment of type 2 diabetes and has pleiotropic antioxidant and anti-inflammatory effects. The aim of this study was to evaluate the neuroprotective effects of dapagliflozin in an experimental model of streptozotocin (STZ)-induced diabetic peripheral neuropathy in mice. C57BL/6 mice were divided into three groups: control (DM–), STZ-induced diabetes (DM+), and diabetes + dapagliflozin (DM + DAPA, 10 mg/kg/day, oral administration for 12 weeks). Clinical (glycemia, weight, diuresis), electrophysiological, and histopathological parameters were evaluated, and behavioral tests (Open Field, Von Frey, Hot Tail) were performed. Dapagliflozin significantly reduced hyperglycemia, limited weight loss and polyuria, and improved locomotor behavior and nociceptive sensitivity. Electrodiagnostically, the treatment increased the amplitude and reduced the duration of motor potentials, indicating improved nerve conduction. Histological analyses showed decreased hydroxynonenal (HNE) immunoreactivity, suggesting attenuation of oxidative stress, reduced perineural fibrogenesis, and maintained intraepidermal nerve fiber density. Dapagliflozin exerts significant neuroprotective effects in experimental diabetic peripheral neuropathy by reducing oxidative stress, inflammation, and fibrosis and maintaining the structural and functional integrity of peripheral nerves.

## 1. Introduction

Diabetes mellitus (DM) is a chronic metabolic disease characterized by persistent hyperglycemia and impaired pancreatic β-cell function and/or insulin resistance, with systemic consequences at the vascular, renal, hepatic, and nervous levels [1]. In recent years, the global prevalence of diabetes has continued to increase, a situation that amplifies the burden of associated complications, including nephropathy, retinopathy, neuropathy, and cardiovascular disease [2,3].

The experimental model of diabetes induced by STZ administration in mice is widely used to investigate the pathological mechanisms related to β-cell destruction and secondary hyperglycemia. STZ is a cytotoxic agent with affinity for β-cells, which generates necrosis and insulin dysfunction, thus gradually generating a model of type 1 or “cytotoxic” type diabetes, depending on the scheme (dose, number of injections) [4,5]. Furthermore, this model allows for the assessment of metabolic, oxidative–inflammatory, and apoptotic changes induced by hyperglycemia, in addition to testing therapeutic strategies with translational potential [5].

In this paradigm, inhibitors of the sodium–glucose cotransporter 2 (SGLT2) have attracted considerable attention. Dapagliflozin, one such SGLT2 inhibitor, has been approved for clinical therapy of type 2 diabetes, with effects on blood glucose lowering, body weight reduction, and cardiovascular and renal protection [6,7,8]. Furthermore, recent experimental data suggest that dapagliflozin may exert beneficial effects beyond glucose metabolism, including reducing oxidative stress, inflammation, fibrogenesis, and apoptosis in diabetic target organs [9,10,11,12]. Building on these observations, the glucose-independent actions of dapagliflozin appear to be mediated through several interconnected molecular pathways, including activation of the AMPK/SIRT1 pathway, leading to improved mitochondrial function and reduced ROS production; inhibition of NF-κB signaling and downstream pro-inflammatory cytokines; attenuation of TGF-β-mediated fibrogenic pathways; and modulation of apoptotic balance by decreasing pro-apoptotic markers (e.g., Bax) and enhancing anti-apoptotic signaling (e.g., Bcl-2) [9,10,11,12].

In the context of the STZ model, preclinical studies have demonstrated that dapagliflozin administered to diabetic mice or rats can ameliorate renal, cardiac, or hepatic damage through mechanisms involving activation of the 5’ AMP-activated protein kinase (AMPK) pathway, inhibition of nuclear factor kappa-light-chain-enhancer of activated B cells (NF-κB), reductions in reactive oxygen species formation, and improvements in mitochondrial dysfunction [13,14,15].

Despite the progress made, the extension of the beneficial effects of dapagliflozin on metabolic, oxidative, and apoptotic parameters in an experimental model of STZ-induced diabetes in mice remains unclear. In addition, in the current literature, a “classical” preclinical study (in diabetic rats/mice) evaluating dapagliflozin directly on diabetic peripheral neuropathy (DPN) with standard neuropathy endpoints (nerve conduction, intraepidermal fiber density, hyperalgesia, etc.) is lacking. The evidence in this regard is not yet robust and generalized.

Large, long-term clinical trials with a primary hypothesis of “neuropathy” are still limited. In clinical practice, the treatment of diabetic neuropathy remains focused on glycemic control, risk factor control (hypertension, dyslipidemia), symptom relief (pain, paresthesia), and not necessarily on proven disease modifiers, agents improving long-term disease outcomes [16,17,18].

Therefore, the present study aims to investigate the effects of dapagliflozin administration in STZ-induced diabetic mice on glycemic control, clinical profile, oxidative stress, behavioral changes, and possible histopathological changes in peripheral neuropathy.

## 2. Results

### 2.1. Effects of Dapagliflozin on Clinical Parameters

**Figure 1:**
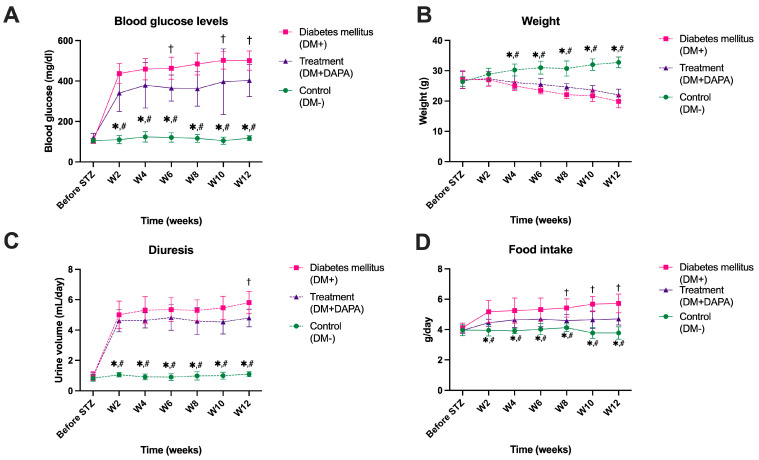
Influence of dapagliflozin on blood glucose (**A**), weight (**B**), diuresis (**C**), and food intake (**D**), both before induction of diabetes mellitus by streptozotocin (STZ) administration and in the following 12 weeks (W). Group of animals with the absence of diabetes—DM–. Group of animals with the presence of diabetes—DM+. Group of animals with the presence of diabetes and treatment with dapagliflozin—DM + DAPA. Each group contained 8 animals. Data are presented as mean ± SD. * *p* < 0.05 vs. DM+, # *p* < 0.05 vs. DP + DAPA, and † *p* < 0.05 vs. DM + DAPA.

After streptozotocin (STZ) administration, blood glucose levels increased significantly in the diabetic group (DM+) compared to the control group (DM–), confirming the onset of experimental diabetes mellitus. Thus, two weeks after diabetes induction, the mean blood glucose levels of mice in the DM+ group reached 437.25 ± 49.46 mg/dL, compared to 110.00 ± 20.35 mg/dL in the control group (*p* < 0.001). During the 12 weeks of monitoring, blood glucose levels in the diabetic group remained consistently high (*p* < 0.001 vs. DM–), reaching 501.25 ± 47.42 mg/dL at the end of the experiment (Figure 1A). Administration of dapagliflozin (DM + DAPA) resulted in a significant reduction in blood glucose compared to no treatment (DM+), with values remaining between 340 and 400 mg/dL throughout the study (*p* < 0.01). Although blood glucose in the treated group remained higher than in the control group, the decreasing trend compared to DM+ highlights a clear antihyperglycemic effect of dapagliflozin (Tables S1–S4).

The evolution of body weight revealed progressive weight loss in untreated diabetic animals (Figure 1B). While control (DM–) mice showed a steady increase in body weight, from 26.37 ± 1.60 g at enrollment to 32.75 ± 1.75 g at week 12, those in the DM+ group showed a significant decrease, from 27.13 ± 2.90 g at enrollment to 19.88 ± 2.10 g (*p* < 0.001). In the dapagliflozin-treated group, body weight remained at intermediate values (22.00 ± 1.93 g at week 12), indicating attenuation of diabetes-induced weight loss. In the dapagliflozin-treated group, body weight showed a discrete, non-significant trend toward reduced weight loss compared to the DM+ group (*p* > 0.05).

Regarding daily urine output (Figure 1C), there was a marked increase in urine volume in untreated diabetic mice, as early as the second week after STZ administration (5.01 ± 0.90 mL/day), with values remaining elevated throughout the experiment (5.81 ± 0.74 mL/day at week 12). In contrast, the control group (DM–) showed stable values, below 1.1 mL/day, without significant changes over time. Treatment with dapagliflozin did not lead to a significant reduction in urine output compared to DM+.

Regarding food intake (Figure 1D), animals in the untreated diabetic group showed a progressive increase in daily intake (from 4.13 ± 0.30 g before STZ to 5.73 ± 0.62 g at week 12), consistent with persistent hyperglycemia and polyphagia specific to diabetes mellitus (*p* < 0.001 vs. DM–). In the dapagliflozin-treated group, mean food intake values were lower (4.70 ± 0.41 g at week 12) and closer to those of controls (3.78 ± 0.41 g), indicating partial amelioration of metabolic disorders. Overall, the data presented in Figure 1A–D confirm the establishment of a stable experimental model of diabetes mellitus, characterized by severe hyperglycemia, weight loss, polyuria, and hyperphagia. Chronic administration of dapagliflozin resulted in a significant improvement in these parameters, reducing glycemia, diuresis, and food intake and limiting weight loss, indicating an overall beneficial effect on the metabolic status of diabetic mice.

### 2.2. Effects of Dapagliflozin on Behavioral Changes

#### 2.2.1. Analysis by Using Open Field Test

**Figure 2:**
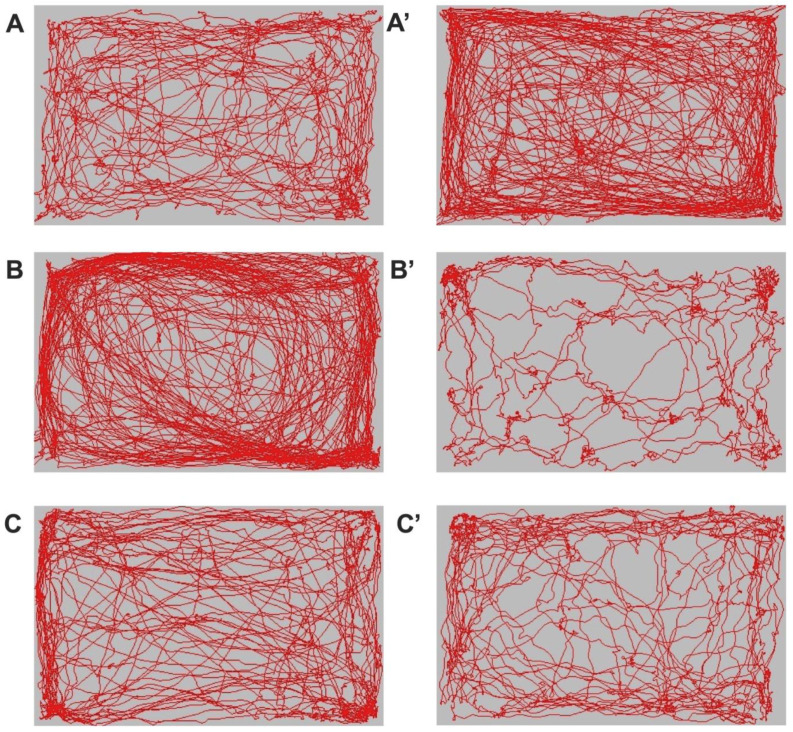
The travel pathways (red) representative of the animals included in the study. (**A**,**A**’)—the group of animals without diabetes mellitus. (**B**,**B**’)—the group of animals with diabetes mellitus without treatment. (**C**,**C**’)—the group of animals with diabetes mellitus and dapagliflozin treatment. (**A**,**B**,**C**)—before streptozotocin administration. (**A**’,**B**’,**C**’)—before euthanasia of the animals.

Analysis of locomotor and exploratory behavior via the Open Field test revealed significant changes between the experimental groups, both in terms of the spatial distribution of trajectories and the density of movements in the central versus peripheral area of the arena. In the representative images shown in Figure 2A,A’, mice in the control group (DM–) exhibited sustained motor activity throughout the experiment, with uniform trajectories covering both the central and peripheral areas of the arena, indicating a balanced exploratory behavior and a reduced level of anxiety. In contrast, untreated diabetic mice (DM+) showed a marked reduction in motor activity, as seen in Figure 2B’. Compared to the image recorded before STZ administration (Figure 2B), at the end of the 12-week period, the trajectories were much narrower, predominantly peripheral, and exploration of the central area was limited. This pattern is characteristic of pronounced anxious behavior and a general decrease in exploratory motivation, correlated with the onset of diabetic neuropathy and impaired locomotor performance. In contrast, diabetic mice treated with dapagliflozin (DM + DAPA) showed an activity intermediate between that of controls (DM–) and that of untreated diabetic mice. After 12 weeks of treatment (Figure 2C’), animals in the DM + DAPA group maintained a movement pattern closer to that of the control group (DM–), with trajectories distributed throughout the arena and an increased presence in the central area compared to the DM+ group. These qualitative observations suggest a protective effect of dapagliflozin on motor function and exploratory behavior, possibly through mechanisms that reduce oxidative stress and maintain peripheral nerve integrity.

**Figure 3:**
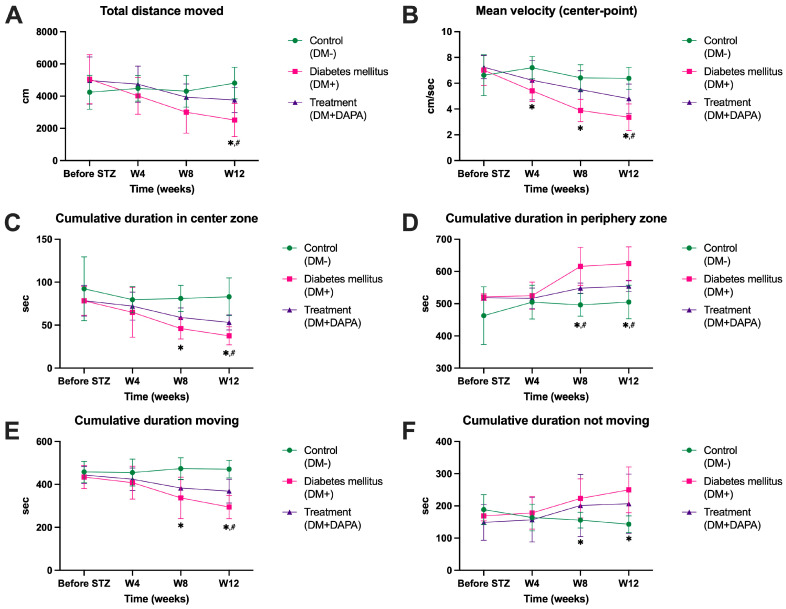
Quantitative analysis of data from the Open Field test. (**A**)—total distance moved. (**B**)—mean velocity (center-point). (**C**)—cumulative duration in the center zone. (**D**)—cumulative duration in the periphery zone. (**E**)—cumulative duration moving. (**F**)—cumulative duration not moving. The group of animals without diabetes—DM–. The group of animals with diabetes—DM+. The group of animals with diabetes and treatment with dapagliflozin—DM + DAPA. Each group contained 8 animals. Data are presented as mean ± SD. * *p* < 0.05 vs. DM+; # *p* < 0.05 vs. DP + DAPA.

Quantitative analysis of the data from the Open Field test confirms the visual observations in Figure 2 and highlights significant differences between the experimental groups. Total distance traveled (Figure 3A) and average speed (Figure 3B) were significantly reduced in animals with untreated diabetes (DM+), especially at 8 and 12 weeks post STZ, compared to the control group (DM–) (*p* < 0.01). Dapagliflozin administration resulted in a significant improvement in these parameters, with the values obtained in the DM + DAPA group being significantly higher than those obtained in the DM+ group at 12 weeks (*p* < 0.05), according to ANOVA followed by Tukey’s post hoc test. Locomotor deficits in DM+ mice showed a very large effect size versus controls (DM–) (d = 2.52), while dapagliflozin improved performance (d = 1.43). Moreover, the mean velocity was markedly reduced in diabetic mice (d = 3.19), with a large improvement after dapagliflozin treatment (d = 1.42). The cumulative time spent in the central area of the arena (Figure 3C) was also reduced in the DM+ group, indicating increased anxious behavior, while animals treated with dapagliflozin spent a significantly longer time in this area at 8 and 12 weeks (*p* < 0.05). Anxiety-like behavior was strongly expressed in DM+ animals (d = 2.56 vs. control), whereas dapagliflozin produced large normalization (d = 1.50). In addition, the cumulative time spent in the peripheral area (Figure 3D) progressively increased in untreated diabetic animals, consistent with the tendency to avoid the center, while the DM + DAPA group presented values close to those of controls (DM–). Time in the periphery increased strongly among DM+ mice (d = 2.29), with dapagliflozin attenuating this effect (d = 1.22). Analysis of total movement duration (Figure 3E) revealed a pronounced decrease among DM+ mice starting from week 8 (*p* < 0.01), and dapagliflozin treatment partially prevented this reduction. Movement duration showed a very large reduction among DM+ mice (d = 3.64), partially corrected by dapagliflozin (d = 1.39). Similarly, the duration of immobility (Figure 3F) was significantly increased in the untreated diabetic group, while in the DM + DAPA group, this parameter was comparable to that of controls (DM–), suggesting an improvement in spontaneous motor capacity. Immobility was substantially increased in diabetic animals (d = 2.23), and dapagliflozin moderately to largely reduced this effect (d = 0.96). Detailed statistical results (ANOVA with Turkey’s correction) confirm these trends: differences between DM+ and DM + DAPA groups were significant at 12 weeks for total distance traveled (*p* = 0.0426), average speed (*p* = 0.0468), duration in the central zone (*p* = 0.0163), duration in the peripheral zone (*p* = 0.0154), duration of movement (*p* = 0.0428), and duration of immobility (*p* = 0.0081). These results indicate that chronic treatment with dapagliflozin attenuates motor and anxiogenic alterations induced by diabetes mellitus, partially restoring normal exploratory behavior.

#### 2.2.2. Assessment of Thermal Hyperalgesia Using Tail Withdrawal Test

**Figure 4:**
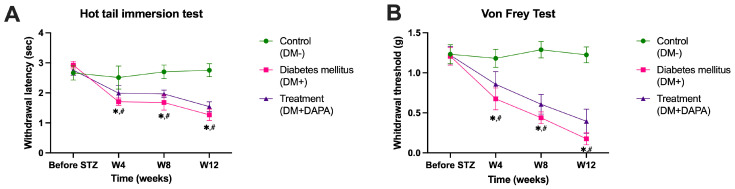
(**A**)—Evaluation of thermal hyperalgesia via tail withdrawal test. (**B**)—Evaluation of mechanical sensitivity and detection of tactile allodynia. Group of animals without diabetes—DM–. Group of animals with diabetes—DM+. Group of animals with diabetes and treatment with dapagliflozin—DM + DAPA. Each group contained 8 animals. Data are presented as mean ± SD. * *p* < 0.05 vs. DM+; # *p* < 0.05 vs. DP + DAPA.

In this study, we assessed thermal hyperalgesia by performing a tail withdrawal test (Figure 4A). At baseline (“Before STZ”), the latency of the tail withdrawal reflex was comparable between all experimental groups, with no statistically significant differences (*p* > 0.05). Starting at week 4 after diabetes induction (W4), the untreated diabetic group (DM+) showed a significant reduction in withdrawal time compared to the control group (DM–) (*p* = 0.0010), indicating the onset of thermal hyperalgesia. This trend was progressively accentuated at weeks 8 and 12 (W8, W12), when the differences between DM+ and DM– were highly significant (*p* < 0.0001). Dapagliflozin (DM + DAPA) administration caused a partial but consistent improvement in the thermal nociceptive response. The latency of the withdrawal reflex in the treated group was significantly higher than in the untreated diabetic group at all evaluation points (W4: *p* = 0.0389; W8: *p* = 0.0381; W12: *p* = 0.0210), although the values did not reach the levels observed in control animals (DM–). At 12 weeks, the differences between the treated and control groups (DM–) remained significant (*p* < 0.0001), suggesting partial restoration of thermal sensitivity under the action of dapagliflozin. Thermal hyperalgesia was pronounced in DM+ mice (d = 6.35 vs. control), while dapagliflozin showed a large improvement (d = 1.28 vs. DM+). These results indicate that antihyperglycemic treatment attenuates diabetes-induced hyperalgesia but does not completely restore normal thermal response.

#### 2.2.3. Evaluation of Mechanical Sensitivity and Detection of Tactile Allodynia

Evaluation of tactile allodynia (Figure 4B) confirmed the installation of progressive mechanical hypersensitivity in diabetic mice. Before the induction of diabetes, the withdrawal threshold was similar between groups (*p* > 0.9). At 4 weeks post STZ, diabetic (DM+) mice showed a significant decrease in withdrawal threshold compared to controls (*p* < 0.0001), a difference that was maintained and amplified at 8 and 12 weeks (*p* < 0.0001).

Treatment with dapagliflozin significantly improved mechanical allodynia, with withdrawal threshold values being higher than those in the DM+ group at all evaluation points (W4: *p* = 0.0679; W8: *p* = 0.0169; W12: *p* = 0.0111). However, compared to the control group, treated animals remained significantly hyposensitive (*p* < 0.001 for W8 and W12). The temporal evolution of the values shows that dapagliflozin reduces the progression of mechanical hypersensitivity induced by chronic hyperglycemia, suggesting a partial neuroprotective effect on peripheral sensory fibers. Mechanical withdrawal thresholds were severely reduced in DM+ mice (d = 9.32 vs. control), with dapagliflozin exerting a large beneficial effect (d = 1.47).

Overall, the data obtained in the behavioral tests confirm the development of painful peripheral neuropathy in untreated diabetic animals, characterized by a decrease in the latency of the nociceptive reflex and the mechanical withdrawal threshold. Chronic administration of dapagliflozin significantly attenuated these alterations, demonstrating a beneficial effect on thermal and mechanical sensitivity, possibly by reducing oxidative stress and improving peripheral microcirculation.

### 2.3. Electrophysiological Evaluation

To evaluate the functional changes associated with diabetic neuropathy and the effect of treatment with dapagliflozin, the electrophysiological parameters of the sciatic nerve—the amplitude and duration of the compound motor potential (CMAP)—were recorded before diabetes induction (Before STZ) and at 4, 8, and 12 weeks post induction.

**Figure 5:**
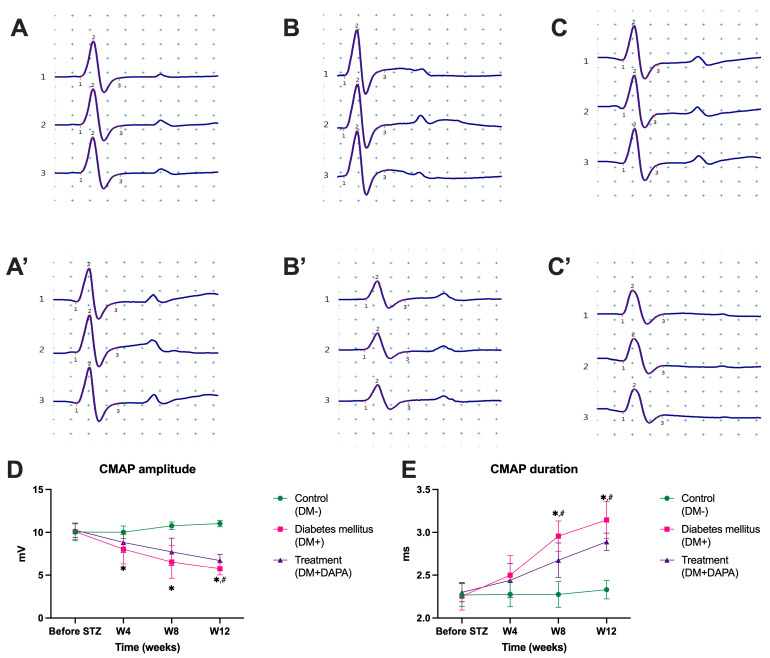
Representative images of compound muscle action potential recordings from the gastrocnemius muscle for sciatic nerve assessment. (**A**,**A**’)—The group of animals without diabetes mellitus. (**B**,**B**’)—The group of animals with diabetes mellitus without treatment. (**C**,**C**’)—The group of animals with diabetes mellitus and dapagliflozin treatment. (**A**,**B**,**C**)—Before streptozotocin administration. (**A**’,**B**’,**C**’)—Before euthanasia of the animals. Evaluation of the amplitude (**D**) and duration (**E**) of the compound muscle action potential (CMAP) for the groups of animals included in the study. The group of animals without diabetes—DM−. The group of animals with diabetes—DM+. The group of animals with diabetes and treatment with dapagliflozin—DM + DAPA. Each group contained 8 animals. Data are presented as mean ± SD. * *p* < 0.05 vs. DM+; # *p* < 0.05 vs. DP + DAPA.

Figure 5 shows representative electromyographic recordings of the compound motor potential (CMAP) obtained before diabetes induction (A–C) and at the end of the 12-week experimental period (A’–C’). Recordings were made at the level of the sciatic nerve, using the same electrical stimulation conditions, to allow for direct comparison of functional changes between groups. In the control group (A, A’), motor potentials showed a typical morphology, characterized by a fast wave, with short latency and high amplitude, reflecting intact nerve conduction and efficient axonal activity. A comparison of recordings made at the beginning and end of the experiment did not reveal notable changes in CMAP amplitude or duration, which confirms the stability of neurophysiological parameters in the absence of diabetes. In contrast, in the untreated diabetic group (B, B’), at the end of the experimental period, a marked reduction in the amplitude of motor potentials and obvious prolongation of the latency were observed, indicating significant impairment of nerve conduction. These changes are suggestive of a mixed type of diabetic neuropathy—axonal and demyelinating—determined by chronic hyperglycemia and associated oxidative stress. The wave morphology became flatter and wider, reflecting a decrease in the number of functional motor fibers and deficient synchronization of the muscle response. In the group treated with dapagliflozin (C, C’), the final recordings revealed partial preservation of the electrophysiological parameters. Although the CMAP amplitude was slightly reduced compared to the initial values, it remained significantly higher than the value recorded for the untreated diabetic group, and the latency of the response showed only minimal prolongation. These results suggest that dapagliflozin treatment exerted a neuroprotective effect, limiting hyperglycemia-induced damage to peripheral nerve conduction.

Overall, the electrophysiological analysis indicates that uncontrolled diabetes mellitus causes a progressive decrease in amplitude and a slowdown in nerve conduction velocity, while chronic administration of dapagliflozin attenuates these changes, contributing to maintaining the functional integrity of the sciatic nerve.

Analysis of the mean values of CMAP amplitude (Figure 5D) revealed progressive differences between the experimental groups over the 12 weeks. In the initial period, before streptozotocin administration (Before STZ), no significant differences were observed between groups (*p* > 0.9), which confirms the functional homogeneity of the groups before diabetes induction. After 4 weeks (W4), animals in the diabetic group (DM+) showed a significant reduction in CMAP amplitude compared to the control group (DM–) (*p* = 0.0349), which indicates the early onset of peripheral nerve dysfunction associated with hyperglycemia. The group treated with dapagliflozin (DM + DAPA) tended to maintain CMAP amplitude at values close to those of the control group, with the differences not being statistically significant (*p* = 0.0786). At 8 weeks (W8), the decrease in CMAP amplitude was more pronounced in untreated diabetic mice compared to both the control (*p* = 0.0008) and treated (*p* = 0.0021) groups, confirming the worsening of motor neuropathy in the absence of treatment. In contrast, the DM + DAPA group showed a significantly higher amplitude compared to the DM+ group (mean difference of 1.188 mV, *p* = 0.3926), suggesting a partial protective effect of dapagliflozin on the functional integrity of the nerve. After 12 weeks (W12), the differences between the groups became marked: CMAP amplitude was significantly lower in untreated diabetic animals compared to the control (mean difference of 5.281 mV, *p* < 0.0001). Dapagliflozin administration caused a significant increase in CMAP amplitude compared to the diabetic group (mean difference of 0.9625 mV, *p* = 0.0472), with the values approaching those of the control group. These results suggest that dapagliflozin attenuated the progressive decrease in the amplitude of the compound motor potential induced by hyperglycemia, confirming a functional neuroprotective effect at the level of the sciatic nerve. The upward trend in the amplitude in the control group, concomitant with the sharp decline in the diabetic group and the relative stabilization in the treated group, reflects the positive impact of the SGLT2 inhibitor on the preservation of the electrical response of motor fibers. At week 12, effect size analysis showed an extremely large reduction in CMAP amplitude in untreated diabetic mice versus controls (Cohen’s d = 9.39), while dapagliflozin produced a large improvement compared to DM+ animals (d = 1.32). This observation is supported by the statistical analysis (ANOVA with post hoc Tukey tests), which demonstrates a significant interaction between the factors “time” and “treatment” (*p* < 0.001).

The analysis of CMAP duration (Figure 5E) completes the picture of peripheral electrical dysfunction. In the prediabetic period (Before STZ), no differences were recorded between the groups (*p* > 0.9999). At 4 weeks (W4), a trend towards an increase in CMAP duration was observed in the diabetic group, although without statistical significance (*p* = 0.0973 compared to the control), suggesting the early onset of nerve conduction changes. Starting at week 8 (W8), the prolongation of CMAP duration became evident and significant: the diabetic group showed a marked increase in duration compared to the control (mean difference of 0.6813 ms, *p* < 0.0001), reflecting the slowing of nerve conduction velocity. The treated group (DM + DAPA) showed a significantly shorter duration compared to DM+ (*p* = 0.0016), suggesting an improvement in motor conduction function under the effect of dapagliflozin. After 12 weeks (W12), the differences between the groups were even more pronounced. Untreated diabetic animals showed a significantly longer CMAP duration compared to the control (*p* < 0.0001), while treatment with dapagliflozin significantly reduced this prolongation (*p* < 0.0001 vs. DM+), maintaining nerve conduction parameters closer to physiological values. CMAP duration markedly increased in the diabetic group (d = 5.27 vs. control), whereas dapagliflozin significantly reduced this prolongation with a very large effect size (d = 2.71).

The parallel evolution of the two variables—the decrease in amplitude and the increase in CMAP duration under hyperglycemia—illustrates the progressive impairment of axonal function and neuronal membrane integrity in diabetic neuropathy. The ameliorating effect of dapagliflozin on both electrophysiological parameters suggests a possible neuroprotective component, which could be correlated with the reduction in oxidative stress and the improvement in neuronal energy metabolism.

### 2.4. Histopathological Analysis

**Figure 6:**
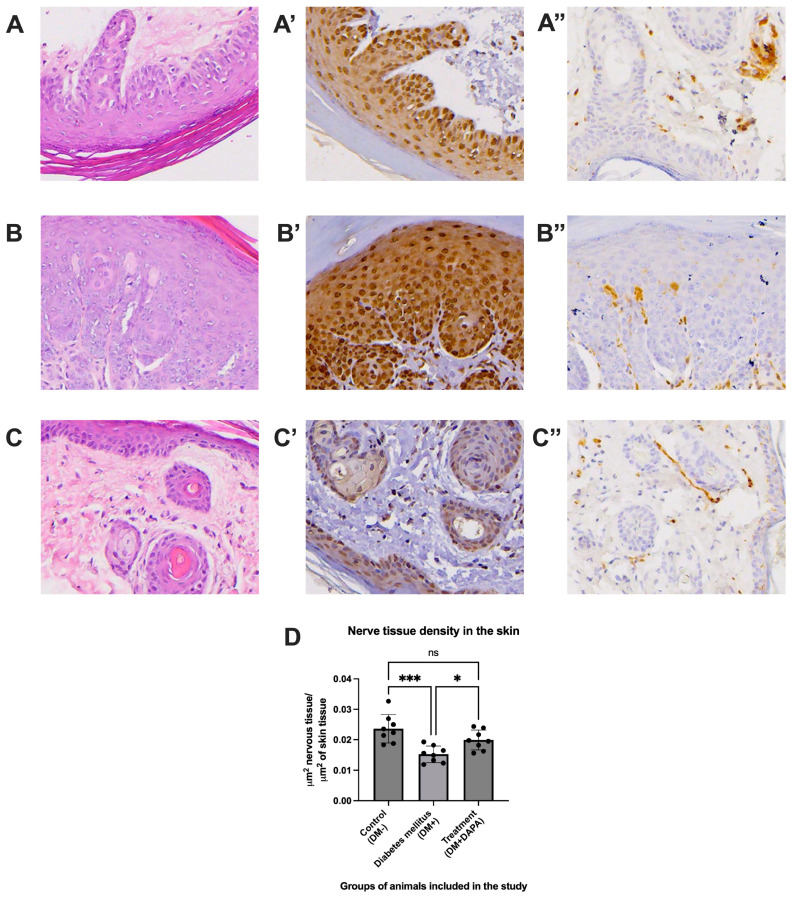
Representative histological images of the plantar dermo-epidermal junction from the animals included in the study. Magnification 20×. (**A**,**A**’,**A**”)—The group of animals without diabetes mellitus. (**B**,**B**’,**B**”)—The group of animals with diabetes mellitus without treatment. (**C**,**C**’,**C**”)—The group of animals with diabetes mellitus and dapagliflozin treatment. (**A**,**B**,**C**)—Hematoxylin–eosin staining. (**A**’,**B**’,**C**’)—4-hydroxynonenal (HNE) staining. (**A**”,**B**”,**C**”)—protein gene product 9.5 (PGP 9.5) staining for the identification of nerve filaments. (**D**)—Evaluation of the density of nerve tissue at the dermo-epidermal junction in the plantar skin of the animals included in the study. The group of animals without diabetes—DM−. The group of animals with diabetes—DM+. The group of animals with diabetes and treatment with dapagliflozin—DM + DAPA. Each group contained 8 animals. Data are presented as mean ± SD. * *p* < 0.05, *** *p* < 0.0005, ns means not significant.

Histological analysis of hematoxylin and eosin (HE)-stained plantar skin sections (Figure 6A–C) revealed marked structural differences between groups. In the control group (DM–), the epithelium presented a normal stratified architecture, with a well-organized epidermis and a compact dermis, without obvious inflammatory changes (Figure 6A). In the diabetic group (DM+), altered tissue organization was observed, with a slightly thickened epidermis, focal cellular degeneration, and a loose dermis, suggesting degenerative changes in the connective components (Figure 6B). Dapagliflozin (DM + DAPA) administration resulted in partial improvement in skin architecture, with partial restoration of dermo-epidermal organization and a reduction in cellular degeneration (Figure 6C). Immunostaining for the oxidative stress marker 4-hydroxynonenal (HNE) (Figure 6A’–C’) revealed a marked increase in HNE expression in the diabetic group, predominantly at the basal layer and suprabasal epithelial cells, indicating an intensification of neuronal and epithelial oxidative stress. In the dapagliflozin-treated group, HNE expression was significantly reduced compared to the DM+ group, suggesting an antioxidant protective effect of the treatment. Immunostaining with protein gene product (PGP) 9.5, used to identify intraepidermal nerve filaments (Figure 6A”–C”), showed a decreased density of nerve endings in diabetic animals, consistent with diabetic peripheral neuropathy. Dapagliflozin treatment was associated with a significant increase in nerve density compared with the DM+ group, indicating potential partial restoration of cutaneous innervation. Quantitative analysis of nerve tissue density (Figure 6D) confirmed the following morphological observations: compared to the control group, diabetic mice showed a significant reduction in the density of cutaneous nerve filaments (*p* < 0.001), and dapagliflozin treatment caused a significant increase in this parameter (*p* < 0.05), according to ANOVA. Cutaneous nerve fiber density was markedly reduced in DM+ animals (d = 2.38), whereas dapagliflozin promoted partial recovery (d = 1.53).

**Figure 7:**
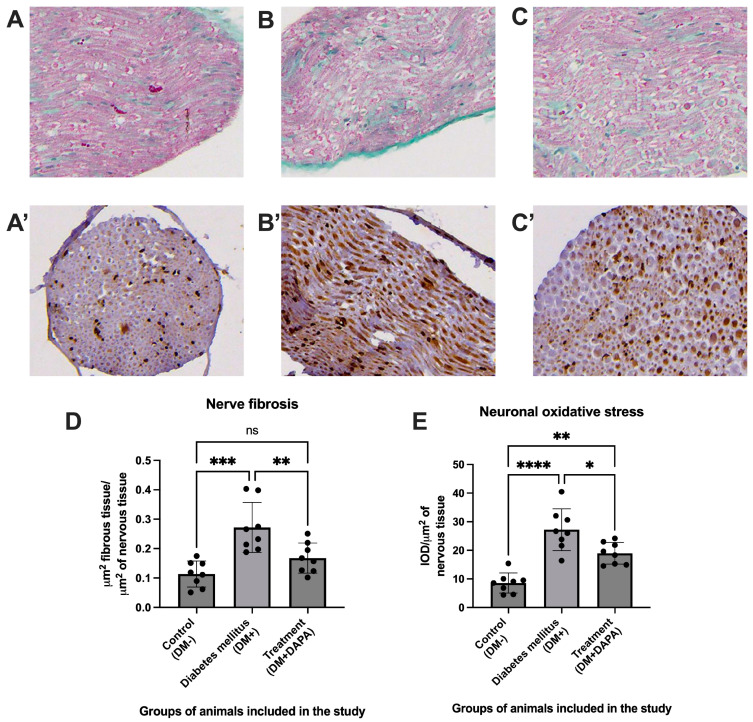
Representative histological images of sciatic nerve sections from animals included in the study. Magnification 20×. (**A**,**A**’)—The group of animals without diabetes mellitus. (**B**,**B**’)—The group of animals with diabetes mellitus without treatment. (**C**,**C**’)—The group of animals with diabetes mellitus and treatment with dapagliflozin. (**A**,**B**,**C**)—Masson trichrome staining. (**A**’,**B**’,**C**’)—4-hydroxynonenal (HNE) staining. (**D**)—The density of fibrous tissue at the level of the sciatic nerve. (**E**)—Evaluation of the degree of oxidative stress at the level of the sciatic nerve. The group of animals without diabetes—DM–. The group of animals with diabetes—DM+. The group of animals with diabetes and treatment with dapagliflozin—DM + DAPA. Each group contained 8 animals. Data are presented as mean ± SD. * *p* < 0.05, ** *p* < 0.005, *** *p* < 0.0005, and **** *p* < 0.0000, ns means not significant.

Masson trichrome staining of sciatic nerve sections (Figure 7A–C) revealed the accumulation of fibrous connective tissue around nerve bundles in diabetic animals, characteristic of fibrogenesis associated with diabetic neuropathy. In the DM+ group, a marked increase in perineural and endoneural collagen deposits was observed, reflected by an increased intensity of the green-blue staining. In contrast, dapagliflozin treatment visibly reduced the extension of these deposits, suggesting a decrease in the process of nerve fibrosis. At the level of the sciatic nerve, HNE staining (Figure 7A’–C’) showed an obvious increase in immunoreactivity for 4-hydroxynonenal in the diabetic group (DM+) compared to controls, suggesting the accumulation of lipid peroxidation products and activation of neuronal oxidative stress. Dapagliflozin significantly reduced the intensity of HNE immunostaining, indicating attenuation of hyperglycemia-induced oxidative stress. Quantitative data (Figure 7D) confirmed the morphological observations: the ratio of fibrous tissue/nerve (µm^2^) was significantly increased in the diabetic group (0.2718 ± 0.08517) compared to controls (0.1135 ± 0.04407; *p* = 0.0002). Dapagliflozin administration significantly reduced the degree of fibrosis (0.1677 ± 0.06133; *p* = 0.0088 vs. DM+), indicating a significant antifibrotic effect. Nerve fibrosis increased substantially in diabetic mice (d = 2.29 vs. controls) and was significantly reduced by dapagliflozin (d = 1.39). Statistical analysis (Figure 7E) confirmed that the mean values of optical intensity (IOD/µm^2^) of HNE were significantly higher in diabetic animals (27.21 ± 3.703) compared to controls (8.553 ± 1.264; *p* < 0.0001). Dapagliflozin administration significantly reduced oxidative stress (18.96 ± 3.805; *p* = 0.0017 vs. DM+), which supports the hypothesis of a neuroprotective effect of SGLT2 inhibitors on neuronal oxidative stress. HNE expression showed a very large increase in DM+ animals (d = 3.43), with dapagliflozin markedly reducing oxidative stress (d = 2.30).

Effect size analysis consistently confirmed the magnitude of the differences observed, with diabetic mice showing large to extremely large impairments across behavioral, electrophysiological, and histological endpoints (Cohen’s d ranging from 2.2 to 9.4), while dapagliflozin produced medium-to-very-large protective effects (d = 0.96–2.71), supporting strong biological relevance beyond *p*-values.

Overall, the histological and morphometric results demonstrate that diabetes mellitus induced by streptozotocin causes major morphological and functional changes at the level of peripheral nerves, characterized by pronounced fibrosis, neuronal oxidative stress, and a loss of density of cutaneous nerve filaments. Dapagliflozin administration exerted notable protective effects, attenuating oxidative stress, reducing fibrogenesis, and promoting partial regeneration of the peripheral nervous network. These results support the hypothesis that SGLT2 inhibitors may have a neuroprotective role in the context of diabetic neuropathy, through mechanisms involving a reduction in oxidative stress and remodeling of the extracellular matrix.

## 3. Discussion

### 3.1. Molecular Mechanisms of Neuronal Damage in Diabetes Mellitus

Diabetic neuropathy is one of the most common and disabling chronic complications of diabetes mellitus, affecting up to 50% of patients [19,20]. Its etiopathogenesis is complex, multifactorial and involves interactions between chronic hyperglycemia, oxidative stress, low-grade inflammation, mitochondrial dysfunction, and microvascular ischemia [21,22,23]. Persistent hyperglycemia leads to activation of the polyol pathway, accumulation of intracellular sorbitol and fructose, and depletion of NADPH, which reduces glutathione regeneration and amplifies oxidative stress [24]. In parallel, activation of the hexosamine pathway and accumulation of advanced glycation end products (AGEs) cause alterations in structural proteins, the stiffening of vessels, and activation of the RAGE receptor, which stimulates the NF-κB cascade and the release of pro-inflammatory cytokines (IL-6, TNF-α) [25,26]. At the neuronal level, mitochondrial oxidative stress generates DNA damage and lipid peroxidation, compromising axonal and synaptic integrity [27,28,29]. Moreover, the reduction in antioxidant enzyme activity (SOD, catalase, GPx) favors the accumulation of reactive oxygen species (ROS) and nitrogen (RNS), and endoneural hypoxia resulting from microvascular dysfunction amplifies nerve damage [30,31]. In addition, dysregulation of axonal transport flux, alterations in NAD^+^ metabolism, and decreased expression of neurotrophic factors (NGF, BDNF) contribute to the progressive degeneration of sensory and motor fibers [32,33]. The experimental model of diabetes induced by streptozotocin (STZ) reproduces these mechanisms, highlighting axonal degeneration, segmental demyelination, and a loss of intraepidermal nerve fiber density [34,35,36].

The data from the present study, specifically the reduction in CMAP amplitude, the prolongation of motor potential latency, the decrease in cutaneous nerve density, and the increase in 4-hydroxynonenal (HNE) expression in untreated diabetic mice, confirm these observations. These changes are congruent with the hypothesis of oxidative stress as a central mechanism of diabetic neuropathy [37].

### 3.2. The Clinical Relevance of Neurodegenerative Pathways and Neuroprotective Therapies Investigated in Diabetic Neuropathy

In the last two decades, numerous therapeutic approaches have been explored to intervene in the pathogenic mechanisms of diabetic neuropathy. Tight glycemic control remains the main determinant of disease progression, but its benefit in peripheral neuropathy is modest, especially in type 2 diabetes [38,39,40,41]. Therefore, current strategies aim to reduce oxidative stress, inflammation, and endothelial dysfunction. Antioxidants such as α-lipoic acid and benfotiamine have shown moderate beneficial effects on sensory symptoms and nerve conduction [42,43,44]. In addition, inhibitors of AGE formation (aminoguanidine, alagebrium) and anti-RAGE agents have been experimentally evaluated, showing reductions in axonal damage and inflammation [45,46,47,48]. Another group of therapies aims to activate AMPK and improve mitochondrial metabolism. Metformin, resveratrol, and sirtuin activators (SIRT1) have shown neuroprotective effects in preclinical models by reducing ROS production and restoring neuronal energy balance [49,50,51,52]. Moreover, anti-inflammatory agents such as NF-κB, IL-1β, or TNF-α inhibitors have reduced neurodegeneration in experimental models [53,54,55]. Recently, neurotrophic factors (NGF, GDNF, IGF-1) and mesenchymal stem cell-based therapies have shown regenerative potential in peripheral nerve fibers [53,54,55,56,57,58,59]. However, the clinical applicability of these therapies remains limited by poor bioavailability, toxicity, or lack of advanced phase studies. Therefore, the identification of agents with multiple actions—metabolic, antioxidant, and anti-inflammatory—represents a research direction of major interest in diabetic neuropathy.

### 3.3. The Effects of Dapagliflozin and Other SGLT2 Inhibitors on the Peripheral Nervous System

Dapagliflozin, a selective inhibitor of sodium–glucose cotransporter type 2 (SGLT2), was initially introduced as an antihyperglycemic agent, but multiple studies have demonstrated significant pleiotropic effects, including cardiovascular, renal, and metabolic protection [60,61,62,63,64]. In recent years, attention has focused on the potential neuroprotective role of SGLT2 inhibitors in diabetes in the context of reducing oxidative stress, inflammation, and fibrogenesis [65,66,67,68].

Several experimental studies have shown that dapagliflozin reduces systemic oxidative stress by activating AMPK and increasing SIRT1 expression, inhibits the NF-κB pathway, and decreases TNF-α and IL-6 levels in insulin-sensitive tissues [69,70,71]. In animal models of diabetes, dapagliflozin has been shown to reduce mitochondrial damage, improve endothelial function, and attenuate renal and cardiac fibrosis [11,72,73,74]. At the neuronal level, SGLT2 inhibitors appear to reduce intracellular glucose accumulation and prevent energy dysfunction and apoptosis of sensory neurons [75]. Recent data suggest that empagliflozin and dapagliflozin can restore corneal and peripheral nerve fiber density, improving electrophysiological parameters and hyperalgesia in preclinical models of neuropathy [76,77].

The results of the present study are congruent with these observations: dapagliflozin significantly reduced glycemia, oxidative stress (decreased HNE), and fibrogenesis (evaluated histologically by Masson staining) and partially restored electrophysiological (CMAP amplitude) and behavioral parameters (Open Field, Von Frey, and Hot Tail tests). These effects can be attributed not only to the reduction in glycemia but also to the direct influence on redox homeostasis and neuronal metabolism.

Possible mechanisms also include activation of AMPK/SIRT1 pathways and inhibition of mitochondrial oxidative stress, as demonstrated by other studies with empagliflozin and canagliflozin [78,79,80]. In addition, SGLT2 inhibition has been suggested to modulate microglial inflammation and the expression of proapoptotic markers (Bax/Bcl-2), preventing axonal degeneration [81,82]. Therefore, our results support the hypothesis that dapagliflozin exerts a complex neuroprotective effect by reducing oxidative stress, inflammation, and fibrogenesis, contributing to the maintenance of the structural and functional integrity of peripheral nerves.

### 3.4. Limitations and Future Directions

The limitations of this study include the small sample size and the limited treatment duration of 12 weeks. Moreover, the molecular expressions of the AMPK/SIRT1/NF-κB pathways and neuronal metabolomic changes were not evaluated. Further studies are needed to confirm the neuroprotective effects of dapagliflozin in chronic models and to explore the clinical applicability of these observations in patients with diabetic neuropathy. The integration of functional, morphological, and molecular data will allow us to achieve a precise definition of the mechanisms involved and validate dapagliflozin as a potential disease-modifying agent in diabetic neuropathy. On the other hand, the Open Field results may be influenced by factors such as anxiety, body weight, and general metabolic status. The Von Frey and Hot Tail tests remain the primary functional neuropathy measures, while Open Field testing provides supporting contextual behavioral information. A major limitation of the present study is the absence of an insulin-treated comparator arm, which would help us to determine whether the neuroprotective effects of dapagliflozin are mediated exclusively by reduced glycemia or by intrinsic pharmacological mechanisms. However, despite persistent hyperglycemia throughout the study, dapagliflozin significantly improved electrophysiological, behavioral, and histological parameters, suggesting at least a partial glucose-independent mechanism, consistent with previous research. Future studies are warranted that include insulin-treated control groups so we can better discriminate glycemic effects from direct neuroprotective actions.

Although our study only covers a 12-week treatment period, existing evidence suggests that long-term administration of SGLT2 inhibitors maintains antioxidant, anti-inflammatory, and antifibrotic activity without major neurotoxic effects [65,66,67,68]. Nevertheless, chronic treatment may induce osmotic diuresis, volume depletion, or mild weight loss, and these aspects warrant dedicated evaluation in long-duration DPN models [76,77]. Future studies will assess whether sustained modulation of AMPK/SIRT1 and mitochondrial pathways translates into lasting structural and functional nerve protection.

## 4. Materials and Methods

### 4.1. Animal Experiments

This study was carried out using an animal model with DM. From a structural and functional point of view, we analyzed the changes produced in diabetic neuropathy. This study was analytical, experimental, and prospective. All institutional guidelines for experiments on laboratory animals were respected, and approval was obtained from the Ethics Committee of the University of Medicine and Pharmacy (UMF) of Craiova (no 36/20 January 2023). The UMF animal facility, where our experiment was conducted, operates under FELASA (Federation of Laboratory Animal Science Association) accreditation. All procedures involving animals comply with the Standards Relating to the Care and Management of Experimental Animals in research and other regulations. This study complies with the relevant national, EU, and international ethics-related rules and professional codes of conduct. The experiments were performed in accordance with the European Council Directive (86⁄609⁄EEC). Moreover, the pathological assessment was performed in the Research Center for Microscopic Morphology and Immunology studies at the UMF of Craiova.

In this study, we used 24 male C57BL/6 mice, aged between 8 and 10 weeks, weighing between 18 and 28 g, housed in a pathogen-free environment with continuous access to food and water and a 12 h light/12 h dark schedule. Before the procedures took place, the animals were removed from the animal care complex. Plasma glucose was determined at enrollment (before the induction of DM) and every week after disease onset, enabling us to diagnose and measure induction duration. To measure plasma glucose, we collected blood from a large tail vein using a standard glucometer (Contour Plus One, Ascensia Diabetes care, Basel, Switzerland). For the analysis of diuresis and the amount of food ingested per day, we used metabolic cages.

To induce DM, a single dose of 150 mg/kg body weight of STZ was intraperitoneally injected. STZ-induced diabetes was confirmed within 72 h after injection.

The animals were randomly divided into three distinct groups (8 animals per group), as follows:

1. The control group (DM–) that received NaCl 0.9%, without STZ. Diabetes mellitus did not occur in this group of animals. In this group, we evaluated the clinical changes (body weight, blood glucose, diuresis, food intake), as well as the changes in the sciatic nerve and the nerve fibers in the skin in the absence of DM.
2. The diabetes mellitus group (DM+) which received a single dose of STZ, 150 mg/kg body weight, injected intraperitoneally. The animals in this group did not receive any therapeutic protocol. The biological parameters of the animals were also evaluated (body weight, blood glucose, diuresis, food intake), as were the structural and functional parameters of the sciatic nerve and the nerve fibers in the skin.
3. The treatment group (DM+ plus treatment with dapagliflozin—DM + DAPA). In the case of these animals, after the induction of DM by intraperitoneal injection of STZ and confirmation of the presence of diabetes according to repeated elevated blood glucose levels, the therapeutic protocol was applied by administering 10 mg dapagliflozin/kg body weight via gastric gavage. Dapagliflozin treatment was started after confirmation of persistent hyperglycemia. It was prepared fresh daily immediately before administration, dissolved in 0.9% saline, and delivered via oral gavage. No stock solutions were stored. Treatment was applied daily for 12 weeks.

The 10 mg/kg/day dose was selected because it is the most widely used preclinical dose in mouse models of diabetic complications [11,13,72,73]. We used the FDA’s body surface area conversion formula, HED (mg/kg) = 10 × (3/37) = 0.81 mg/kg, which corresponds to approximately 57 mg/day for a 70 kg adult [83]. Considering rodents’ higher metabolic rate and faster clearance, this dose is pharmacologically aligned with the clinically approved 10 mg/day dose in humans.

### 4.2. Behavioral Tests

#### 4.2.1. Open Field Test

The evaluation of exploratory behavior and the degree of anxiety was performed using the Open Field test in mice. The tests were performed at four distinct times: before the initiation of treatment (baseline, before STZ), 4 weeks after the start of STZ administration, and 8 weeks and 12 weeks immediately prior to animal sacrifice.

The experiments were conducted in a test room with reduced ambient lighting, controlled temperature (22 ± 1 °C), and minimal ambient noise. The animals were transferred to the test room at least 30 min before the start of the test for acclimatization.

The test arena was made of light-colored plexiglass, measuring 50 cm (length) × 33 cm (width) × 15 cm (height). The surface was virtually delimited into two areas of interest: the central area and the peripheral area. Each mouse was placed individually in the center of the arena and allowed to move freely for 10 min, and their behavior was recorded with a video camera mounted above the arena.

The analyzed parameters included average movement speed (cm/s), total distance traveled (cm), time spent in the central area (s), and time spent in the peripheral area (s). The recordings were processed with EthoVision XT 14 software (Version 14, Noldus Information Technology, Wageningen, The Netherlands) for automatic determination of kinetic and spatial parameters.

Between each experimental session, the arena was cleaned with 70% ethanol and dried completely to eliminate olfactory traces and prevent subsequent subjects being influenced.

#### 4.2.2. Von Frey Test

To determine tactile allodynia in mice, the Von Frey test was used, considered a standardized method for evaluating mechanical sensitivity. The animals were placed individually in a cage with a stainless steel mesh bottom and allowed to acclimate for at least 15 min with the aim of reducing behavioral stress and stabilizing reflex responses. Subsequently, a series of calibrated Von Frey filaments (different force ranges measured in g) were progressively applied, vertically, to the left hind paw of each specimen until slight flexion of the filament was obtained.

The observed behavioral reactions—such as raising, shaking, or licking their paw—were considered positive responses, indicating the presence of increased nociceptive sensitivity. Each animal was tested three times, maintaining a minimum interval of 10 min between determinations, and the individual values were subsequently aggregated by calculating the arithmetic mean.

The Von Frey test is widely used to assess mechanical sensitivity and detect tactile allodynia in experimental models of neuropathic or inflammatory pain. This method allows for the determination of the threshold of response to tactile stimuli via the controlled application of mechanical forces of variable intensity to the skin surface.

#### 4.2.3. Hot Tail Immersion Test

The hot water tail immersion test is a standardized experimental method used to evaluate thermal hyperalgesia in rodents, especially mice. This technique allows for the assessment of thermal pain sensitivity by measuring the latency of the tail withdrawal reflex to a controlled thermal stimulus.

In the experiment, a container of water maintained at a constant temperature of 50 ± 0.5 °C was prepared, ensuring thermal homogeneity by means of gentle agitation and continuous monitoring with a precision digital thermometer. Each animal was handled with care to reduce stress, being lightly immobilized in a soft towel so that only the tail remained exposed.

Approximately one-third of the distal length of the tail was quickly immersed in water, and the timer was started at the moment of contact. The time elapsed until the tail flick reflex was recorded, which represents the animal’s physiological response to the painful stimulus.

To prevent possible tissue damage caused by prolonged exposure to heat, a maximum exposure time of 30 s was established, after which the tail was removed even if the reflex was not observed.

Each mouse was subjected to four consecutive experimental tests in order to obtain reproducible data and reduce individual variations. An interval of 5 min was maintained between two successive determinations, the time necessary to restore basal temperature and avoid sensitization or adaptation to the stimulus.

The values obtained for each test were recorded individually, and the final result was calculated as the arithmetic mean of the four measurements performed for each animal. This approach allows for an objective and precise assessment of the thermal pain threshold, providing relevant data for pharmacological or physiological studies on nociceptive mechanisms.

### 4.3. Tissue Preparation and Histological Analysis

After euthanasia of the animals under deep anesthesia, sciatic nerves and plantar skin tissue were collected. Subsequently, these tissues were fixed in 4% formalin for 24 h. After 24–48 h of fixation, the tissues were washed for 24 h in order to remove the formalin fixative solution. and then embedded in paraffin. After obtaining the tissue blocks, serial sections of 4 μm thickness were cut using a high-precision HM355S automatic rotary microtome, equipped with a section transfer system on a cold water bath, and subsequently, they were transferred to a hot water bath at a temperature of 40 °C to enable them to stretch and to ensure that they were uniform. The obtained sections were then collected and mounted on poly-L-lysine slides, then placed in an incubator at 60 °C and kept for 24 h. Initially, the tissues were first stained using the hematoxylin–eosin (HE) technique. The sections were sequentially incubated with the primary antibody PGP 9.5 (protein gene product 9.5) for nervous system analysis (Abcam, dilution 1:200), as shown in previous studies [11,84]. Subsequently, they were incubated with HRP-conjugated secondary antibodies (Abcam); for color visualization, diaminobenzidine was used as a chromogenic substrate, followed by hematoxylin counterstaining. Initially, all slides were assessed on a Nikon 55i microscope (Apidrag, Bucharest, Romania), equipped with a 5-megapixel color cooled CCD camera and Image ProPlus AMS 9 software (Media Cybernetics, Rockville, MD, USA). Subsequently, all slides were scanned with a microscope equipped with a MoticEasyScan Pro 6 scanner (Kowloon, Hong Kong) using a 20× objective and digitized using EasyScanner software (version 6). Quantitative analysis of nerve fibers from plantar tissue or the sciatic nerve was performed using Image ProPlus AMS 9 software, with the entire tissue section scanned. Subsequently, the color channel for the nerve tissue was selected, and the area for this color channel as well as the integrated optical density (IOD) were calculated. These values were compared to the values for the total area of the section. In a separate step, the atrioventricular node was manually delimited and its area was calculated, followed by calculation of the IOD. To oxidative stress, HNE (4-hydroxynonenal) expression was determined.

### 4.4. Electrophysiological Monitoring

To analyze the electrophysiological properties, we recorded the CMAP (compound muscle action potential) and calculated the duration and amplitude. When a motor nerve is electrically stimulated, the impulse is transmitted through its fibers to the muscle it innervates [85,86]. The muscle responds by contracting, and this combined electrical muscle activity (the result of the activation of all muscle fibers innervated by that nerve) is known as the CMAP. The amplitude—expressed in millivolts (mV)—shows how many muscle fibers are activated.

A decreased amplitude indicates axonal loss (fewer functional nerve fibers).

A longer duration indicates slower impulse conduction velocity, suggesting demyelination (damage to the myelin sheath). For the sciatic nerve in mice, the CMAP is recorded from the muscle innervated by the sciatic nerve, i.e., usually from one of the hind paw muscles, where motor projections are clear and accessible. In our study, we recorded the CMAP from the gastrocnemius muscle.

Electrophysiological monitoring was performed using a Neuro MEB-4 electromyography system (Neurosoft Ltd., Ivanovo, Russia), equipped with dedicated software. During the experiments, the level of anesthesia (performed with a cocktail of Ketamine and Xylazine) applied to the animals was checked based on pinch reflex to ensure adequate anesthesia and the absence of nociceptive responses. To prevent corneal drying during the procedures, Bepanthen ophthalmic ointment (Bayer, Leverkusen, Germany) was applied to the eyes of the animals. Electroneurographic recordings were performed using four monopolar needle electrodes (SEI EMG, Milan, Italy). Two were positioned for nerve stimulation, and the other two for recording electrical responses at the level of the analyzed nerve. The acquisition parameters were set so that the frequency band was between 20 Hz and 10 kHz, the signal sweep speed was set at 1 ms/division, and recording sensitivity was 5 mV/division. Three electrical stimuli were applied to each animal, each with a duration of 0.2 ms. The intensity of the applied current was 5 mA for the first stimulation, it was increased to 6 mA for the next two if the amplitude of the compound motor potential (CMAP) did not show a significant increase. In two isolated cases, to obtain the maximal motor response, an intensity of 7 mA was used. For each experimental subject, the final analyzed parameters were represented by the average of the maximum values of CMAP amplitude and duration obtained through these stimulations. Before the induction of experimental diabetes by streptozotocin (STZ) administration, all animals underwent baseline electroneurographic measurements and recordings in order to establish baseline control values.

### 4.5. Statistical Analysis

The data from our study were expressed as the mean and standard deviation. We first entered the data into Microsoft Office Excel (Microsoft Corporation, version 2510, Redmond, WA, USA). After being graphically represented, the data were exported to GraphPad software (Version 9.0, San Diego, CA, USA) for statistical analysis. We used the ANOVA test to analyze statistical differences between the means of more than two data groups. One-way ANOVA was used for histological/morphometric parameters, while two-way ANOVA was used for repeated-measures data (glycemia, weight, diuresis, behavioral tests, CMAP). Tukey’s multiple comparisons post hoc test was applied for between-group analyses. Effect sizes (Cohen’s d) and their confidence intervals were calculated. A *p* value < 0.05 was considered to indicate a statistically significant difference between the means of the compared groups. Moreover, *p* < 0.05, 0.01, 0.001, and 0.0001 represent a statistically significant, highly, and very highly significant difference and are marked in the graph by *, **, ***, or ****.

## 5. Conclusions

This experimental study demonstrates that chronic administration of dapagliflozin exerts significant neuroprotective effects in the streptozotocin-induced diabetic peripheral neuropathy model in mice. Treatment with dapagliflozin attenuated hyperglycemia, reduced oxidative stress and fibrogenesis, and partially restored behavioral and electrophysiological parameters associated with diabetic neuropathy. Morphological analysis revealed a decrease in lipid peroxidation (4-hydroxynonenal expression), a reduction in perineural fibrosis, and an increase in the density of intraepidermal nerve filaments in treated animals, supporting a protective effect on peripheral nerve integrity. These results suggest that beyond its antihyperglycemic action, dapagliflozin confers direct antioxidant and antifibrotic benefits, which may contribute to the functional protection of peripheral nerves. Although further studies are needed in the long term, and with detailed exploration of the molecular mechanisms involved, the data obtained support the potential of dapagliflozin as a promising therapeutic agent capable of modifying the evolution of diabetic peripheral neuropathy.

## Data Availability

The original contributions presented in this study are included in the article/Supplementary Materials. Further inquiries can be directed to the corresponding author.

## Supplementary Materials

The following supporting information can be downloaded at: https://www.mdpi.com/article/10.3390/ijms262412034/s1.

## Abbreviations

The following abbreviations are used in this manuscript:

CMAP	Compound Muscle Action Potential
DAPA	Dapagliflozin
DM	Diabetes Mellitus
HE	Hematoxylin and Eosin
HNE	4-Hydroxynonenal
IOD	Integrated Optical Density
PGP	Protein Gene Product 9.5
