# Supplementary material for: Dapagliflozin Preserves Peripheral Nerve Structure and Reduces Neuropathic Damage in Streptozotocin-Induced Diabetic Peripheral Neuropathy
Source: Int J Mol Sci. 2025 Dec 14;26(24):12034. doi: 10.3390/ijms262412034 (PMC12733237; doi:10.3390/ijms262412034)
Supplement: Supplementary file 1 [file ijms-26-12034-s001.zip › ijms-4011118-supplementary.pdf]

Table S1. 2way ANOVA of blood glucose level

Tukey's multiple comparisons test	Mean diff,	95,00% CI of diff,	Below threshold?	Summary	Adjusted P Value
Before STZ					
Control (DM-) vs. Diabetes mellitus (DM+)	2,125	-13,30 to 17,55	No	ns	0,9312
Control (DM-) vs. Treatment (DM+DAPA)	-13,38	-37,75 to 11,00	No	ns	0,3332
Diabetes mellitus (DM+) vs. Treatment (DM+DAPA)	-15,50	-39,95 to 8,948	No	ns	0,2424
W2					
Control (DM-) vs. Diabetes mellitus (DM+)	-327,3	-379,7 to -274,8	Yes	****	<0,0001
Control (DM-) vs. Treatment (DM+DAPA)	-230,6	-325,5 to -135,8	Yes	***	0,0003
Diabetes mellitus (DM+) vs. Treatment (DM+DAPA)	96,63	-2,443 to 195,7	No	ns	0,0560
W4					
Control (DM-) vs. Diabetes mellitus (DM+)	-334,3	-391,3 to -277,2	Yes	****	<0,0001
Control (DM-) vs. Treatment (DM+DAPA)	-254,6	-372,4 to -136,9	Yes	***	0,0007
Diabetes mellitus (DM+) vs. Treatment (DM+DAPA)	79,63	-41,29 to 200,5	No	ns	0,2169
W6					
Control (DM-) vs. Diabetes mellitus (DM+)	-342,0	-400,1 to -283,9	Yes	****	<0,0001
Control (DM-) vs. Treatment (DM+DAPA)	-244,1	-312,1 to -176,1	Yes	****	<0,0001
Diabetes mellitus (DM+) vs. Treatment (DM+DAPA)	97,88	19,37 to 176,4	Yes	*	0,0148
W8					
Control (DM-) vs. Diabetes mellitus (DM+)	-367,3	-424,4 to -310,1	Yes	****	<0,0001
Control (DM-) vs. Treatment (DM+DAPA)	-245,1	-335,3 to -155,0	Yes	***	0,0002
Diabetes mellitus (DM+) vs. Treatment (DM+DAPA)	122,1	25,72 to 218,5	Yes	*	0,0141
W10					
Control (DM-) vs. Diabetes mellitus (DM+)	-396,9	-443,0 to -350,7	Yes	****	<0,0001
Control (DM-) vs. Treatment (DM+DAPA)	-291,3	-460,3 to -122,2	Yes	**	0,0034
Diabetes mellitus (DM+) vs. Treatment (DM+DAPA)	105,6	-64,16 to 275,4	No	ns	0,2371
W12					
Control (DM-) vs. Diabetes mellitus (DM+)	-383,8	-433,3 to -334,2	Yes	****	<0,0001
Control (DM-) vs. Treatment (DM+DAPA)	-285,0	-368,4 to -201,6	Yes	****	<0,0001
Diabetes mellitus (DM+) vs. Treatment (DM+DAPA)	98,75	10,30 to 187,2	Yes	*	0,0290

Table S2. Row statistics of blood glucose level

		Control (DM-)			Diabetes mellitus (DM+)			Treatment (DM+DAPA)		
Before STZ		105,25	11,6465813500296	8	103,125	11,9216190175664	8	118,625	22,513091429528	[illegible]
W2		110	20,3540097839643	8	437,25	49,4592184098605	8	340,625	90,8938745382296	[illegible]
W4		124,375	25,5339409973694	8	458,625	53,071212266431	8	379	112,801975666589	[illegible]
W6		121,125	23,1913623082881	8	463,125	54,6480623104398	8	365,25	64,5417915551068	[illegible]
W8		116,75	19,6813907754798	8	484	54,1664102558034	8	361,875	86,3604365435933	[illegible]
W10		105,375	17,7919524344174	8	502,25	43,5356996367021	8	396,625	162,331176039249	[illegible]
W12		117,5	12,4326758411626	8	501,25	47,4213634798254	8	402,5	80,0838845925232	[illegible]

Table S3. 2way ANOVA of body weigh

Tukey's multiple comparisons test	Mean diff,	95,00% CI of diff,	Below threshold?	Summary	Adjusted P Value
Before STZ					
Control (DM-) vs. Diabetes mellitus (DM+)	-0,7500	-3,917 to 2,417	No	ns	0,8013
Control (DM-) vs. Treatment (DM+DAPA)	-0,5000	-3,573 to 2,573	No	ns	0,9003
Diabetes mellitus (DM+) vs. Treatment (DM+DAPA)	0,2500	-3,481 to 3,981	No	ns	0,9832
W2					
Control (DM-) vs. Diabetes mellitus (DM+)	1,875	-0,8109 to 4,561	No	ns	0,1967
Control (DM-) vs. Treatment (DM+DAPA)	1,500	-1,370 to 4,370	No	ns	0,3812
Diabetes mellitus (DM+) vs. Treatment (DM+DAPA)	-0,3750	-3,344 to 2,594	No	ns	0,9416
W4					
Control (DM-) vs. Diabetes mellitus (DM+)	5,250	2,925 to 7,575	Yes	***	0,0001
Control (DM-) vs. Treatment (DM+DAPA)	4,125	1,405 to 6,845	Yes	**	0,0038
Diabetes mellitus (DM+) vs. Treatment (DM+DAPA)	-1,125	-3,604 to 1,354	No	ns	0,4723
W6					
Control (DM-) vs. Diabetes mellitus (DM+)	7,500	5,161 to 9,839	Yes	****	<0,0001
Control (DM-) vs. Treatment (DM+DAPA)	5,500	2,789 to 8,211	Yes	***	0,0003
Diabetes mellitus (DM+) vs. Treatment (DM+DAPA)	-2,000	-4,212 to 0,2124	No	ns	0,0777
W8					
Control (DM-) vs. Diabetes mellitus (DM+)	8,625	5,908 to 11,34	Yes	****	<0,0001
Control (DM-) vs. Treatment (DM+DAPA)	6,125	3,450 to 8,800	Yes	***	0,0002
Diabetes mellitus (DM+) vs. Treatment (DM+DAPA)	-2,500	-4,172 to -0,8285	Yes	**	0,0042
W10					
Control (DM-) vs. Diabetes mellitus (DM+)	10,25	7,740 to 12,76	Yes	****	<0,0001
Control (DM-) vs. Treatment (DM+DAPA)	8,375	6,097 to 10,65	Yes	****	<0,0001
Diabetes mellitus (DM+) vs. Treatment (DM+DAPA)	-1,875	-4,139 to 0,3887	No	ns	0,1112
W12					
Control (DM-) vs. Diabetes mellitus (DM+)	12,88	10,33 to 15,42	Yes	****	<0,0001
Control (DM-) vs. Treatment (DM+DAPA)	10,75	8,337 to 13,16	Yes	****	<0,0001
Diabetes mellitus (DM+) vs. Treatment (DM+DAPA)	-2,125	-4,765 to 0,5149	No	ns	0,1242

Table S4. Row statistics of body weigh

		Control (DM-)			Diabetes mellitus (DM+)			Treatment (DM+DAPA)		
Before STZ		26,375	1,59798980865694	8	27,125	2,90012315009454	8	26,875	2,79987244607423	[illegible]

W2		28,875	1,95940953204931	8	27	2,1380899352994	8	27,375	2,38671920665766
W4		30,25	1,98206241793023	8	25	1,51185789203691	8	26,125	2,16712449375401
W6		31	2,1380899352994	8	23,5	1,19522860933439	8	25,5	2
W8		30,75	2,49284690951645	8	22,125	1,35620268186054	8	24,625	1,18773493916542
W10		32	1,92724822331886	8	21,75	1,90862703084106	8	23,625	1,50594061730772
W12		32,75	1,75254916376933	8	19,875	2,10017006114131	8	22	1,92724822331886

Table S5. 2way ANOVA of diuresis

Tukey's multiple comparisons test	Mean diff,	95,00% CI of diff,	Below threshold?	Summary	Adjusted P Value
Before STZ					
Control (DM-) vs. Diabetes mellitus (DM+)	-0,1375	-0,4854 to 0,2104	No	ns	0,5642
Control (DM-) vs. Treatment (DM+DAPA)	-0,1375	-0,4494 to 0,1744	No	ns	0,4980
Diabetes mellitus (DM+) vs. Treatment (DM+DAPA)	0,000	-0,3596 to 0,3596	No	ns	>0,9999
W2					
Control (DM-) vs. Diabetes mellitus (DM+)	-3,950	-4,893 to -3,007	Yes	****	<0,0001
Control (DM-) vs. Treatment (DM+DAPA)	-3,550	-4,311 to -2,789	Yes	****	<0,0001
Diabetes mellitus (DM+) vs. Treatment (DM+DAPA)	0,4000	-0,6811 to 1,481	No	ns	0,6054
W4					
Control (DM-) vs. Diabetes mellitus (DM+)	-4,388	-5,320 to -3,455	Yes	****	<0,0001
Control (DM-) vs. Treatment (DM+DAPA)	-3,713	-4,243 to -3,182	Yes	****	<0,0001
Diabetes mellitus (DM+) vs. Treatment (DM+DAPA)	0,6750	-0,3032 to 1,653	No	ns	0,1951
W6					
Control (DM-) vs. Diabetes mellitus (DM+)	-4,438	-5,256 to -3,619	Yes	****	<0,0001
Control (DM-) vs. Treatment (DM+DAPA)	-3,913	-4,794 to -3,031	Yes	****	<0,0001
Diabetes mellitus (DM+) vs. Treatment (DM+DAPA)	0,5250	-0,5397 to 1,590	No	ns	0,4228
W8					
Control (DM-) vs. Diabetes mellitus (DM+)	-4,313	-5,050 to -3,575	Yes	****	<0,0001
Control (DM-) vs. Treatment (DM+DAPA)	-3,600	-4,527 to -2,673	Yes	****	<0,0001
Diabetes mellitus (DM+) vs. Treatment (DM+DAPA)	0,7125	-0,3356 to 1,761	No	ns	0,2104
W10					
Control (DM-) vs. Diabetes mellitus (DM+)	-4,463	-5,262 to -3,663	Yes	****	<0,0001
Control (DM-) vs. Treatment (DM+DAPA)	-3,538	-4,368 to -2,707	Yes	****	<0,0001
Diabetes mellitus (DM+) vs. Treatment (DM+DAPA)	0,9250	-0,09350 to 1,943	No	ns	0,0775
W12					
Control (DM-) vs. Diabetes mellitus (DM+)	-4,713	-5,483 to -3,942	Yes	****	<0,0001
Control (DM-) vs. Treatment (DM+DAPA)	-3,700	-4,311 to -3,089	Yes	****	<0,0001
Diabetes mellitus (DM+) vs. Treatment (DM+DAPA)	1,013	0,1371 to 1,888	Yes	*	0,0233

Table S6. Row statistics of diuresis

		Control (DM-)			Diabetes mellitus (DM+)			Treatment (DM+DAPA)	
Before STZ		0,8375	0,226384628453435	8	0,975	0,296407056017806	8	0,975	0,249284690951645
W2		1,0625	0,150594061730772	8	5,0125	0,904650682386775	8	4,6125	0,729848320836988
W4		0,925	0,198206241793023	8	5,3125	0,893528319161098	8	4,6375	0,501248441394086
W6		0,9125	0,223207142742854	8	5,35	0,781938981908801	8	4,825	0,843038721361176
W8		0,9875	0,274837614393871	8	5,3	0,696932052437169	8	4,5875	0,883883476483185
W10		1	0,226778683805536	8	5,4625	0,763333853191755	8	4,5375	0,792712341989739
W12		1,1	0,169030850945703	8	5,8125	0,737636185973236	8	4,8	0,58309518948453

Table S7. 2way ANOVA of food intake

Tukey's multiple comparisons test	Mean diff,	95,00% CI of diff,	Below threshold?	Summary	Adjusted P Value
Before STZ					
Control (DM-) vs. Diabetes mellitus (DM+)	-0,1625	-0,6061 to 0,2811	No	ns	0,6114
Control (DM-) vs. Treatment (DM+DAPA)	0,01250	-0,3966 to 0,4216	No	ns	0,9963
Diabetes mellitus (DM+) vs. Treatment (DM+DAPA)	0,1750	-0,1740 to 0,5240	No	ns	0,4066
W2					
Control (DM-) vs. Diabetes mellitus (DM+)	-1,225	-2,017 to -0,4331	Yes	**	0,0043
Control (DM-) vs. Treatment (DM+DAPA)	-0,5000	-0,9171 to -0,08290	Yes	*	0,0196
Diabetes mellitus (DM+) vs. Treatment (DM+DAPA)	0,7250	-0,04372 to 1,494	No	ns	0,0637
W4					
Control (DM-) vs. Diabetes mellitus (DM+)	-1,338	-2,203 to -0,4715	Yes	**	0,0059
Control (DM-) vs. Treatment (DM+DAPA)	-0,7250	-1,253 to -0,1967	Yes	*	0,0103
Diabetes mellitus (DM+) vs. Treatment (DM+DAPA)	0,6125	-0,3072 to 1,532	No	ns	0,2169
W6					
Control (DM-) vs. Diabetes mellitus (DM+)	-1,300	-2,115 to -0,4853	Yes	**	0,0035
Control (DM-) vs. Treatment (DM+DAPA)	-0,6625	-1,243 to -0,08170	Yes	*	0,0253
Diabetes mellitus (DM+) vs. Treatment (DM+DAPA)	0,6375	-0,2172 to 1,492	No	ns	0,1574
W8					
Control (DM-) vs. Diabetes mellitus (DM+)	-1,300	-1,937 to -0,6626	Yes	***	0,0007
Control (DM-) vs. Treatment (DM+DAPA)	-0,4625	-0,9119 to -0,01310	Yes	*	0,0436
Diabetes mellitus (DM+) vs. Treatment (DM+DAPA)	0,8375	0,1587 to 1,516	Yes	*	0,0163
W10					
Control (DM-) vs. Diabetes mellitus (DM+)	-1,900	-2,479 to -1,321	Yes	****	<0,0001
Control (DM-) vs. Treatment (DM+DAPA)	-0,8750	-1,512 to -0,2380	Yes	**	0,0085
Diabetes mellitus (DM+) vs. Treatment (DM+DAPA)	1,025	0,3195 to 1,731	Yes	**	0,0052
W12					
Control (DM-) vs. Diabetes mellitus (DM+)	-1,950	-2,651 to -1,249	Yes	****	<0,0001
Control (DM-) vs. Treatment (DM+DAPA)	-0,9250	-1,459 to -0,3906	Yes	**	0,0013
Diabetes mellitus (DM+) vs. Treatment (DM+DAPA)	1,025	0,3223 to 1,728	Yes	**	0,0056

Table S8. Row statistics of food intake

		Control (DM-)	Diabetes mellitus (DM+)	Treatment (DM+DAPA)

Before STZ		3,9625	0,370086862390825	8	4,125	0,301188123461543	8	3,95	0,220389266007736	
W2		3,95	0,374165738677394	8	5,175	0,732412842206205	8	4,45	0,232992949004287	
W4		3,9125	0,180772153354911	8	5,25	0,829802041797577	8	4,6375	0,501248441394086	
W6		4,025	0,369362384967083	8	5,325	0,75734498649653	8	4,6875	0,49982139667228	
W8		4,125	0,254950975679639	8	5,425	0,599404466354493	8	4,5875	0,401559460105226	
W10		3,775	0,357571171736681	8	5,675	0,503558763771845	8	4,65	0,570713838726805	
W12		3,775	0,406201920231798	8	5,725	0,622781777878209	8	4,7	0,41057451037714	

Table S9. 2way ANOVA of Total Distance Moved

Tukey's multiple comparisons test	Mean diff,	95,00% CI of diff,	Below threshold?	Summary	Adjusted P Value
Before STZ					
Control (DM-) vs. Diabetes mellitus (DM+)	-811,2	-2551 to 928,1	No	ns	0,4539
Control (DM-) vs. Treatment (DM+DAPA)	-727,0	-2418 to 964,2	No	ns	0,5092
Diabetes mellitus (DM+) vs. Treatment (DM+DAPA)	84,23	-1871 to 2040	No	ns	0,9930
W4					
Control (DM-) vs. Diabetes mellitus (DM+)	467,5	-836,3 to 1771	No	ns	0,6192
Control (DM-) vs. Treatment (DM+DAPA)	-260,3	-1547 to 1026	No	ns	0,8551
Diabetes mellitus (DM+) vs. Treatment (DM+DAPA)	-727,8	-2207 to 751,4	No	ns	0,4246
W8					
Control (DM-) vs. Diabetes mellitus (DM+)	1303	-226,1 to 2832	No	ns	0,0997
Control (DM-) vs. Treatment (DM+DAPA)	372,8	-824,7 to 1570	No	ns	0,6986
Diabetes mellitus (DM+) vs. Treatment (DM+DAPA)	-929,9	-2387 to 527,4	No	ns	0,2435
W12					
Control (DM-) vs. Diabetes mellitus (DM+)	2293	980,2 to 3606	Yes	**	0,0012
Control (DM-) vs. Treatment (DM+DAPA)	1048	-123,8 to 2221	No	ns	0,0823
Diabetes mellitus (DM+) vs. Treatment (DM+DAPA)	-1245	-2450 to -40,09	Yes	*	0,0426

Table S10. Row statistics of Total Distance Moved

		Control (DM-)			Diabetes mellitus (DM+)			Treatment (DM+DAPA)		
Before STZ		4240,78625	1057,81878103601	8	5052,02625	1522,21719785823	8	4967,795	1465,071131925	8
W4		4485,05375	794,014907523557	8	4017,54375	1140,50651108478	8	4745,33125	1119,89087740646	8
W8		4307,01125	992,694566786172	8	3004,31	1303,92879475836	8	3934,21125	822,308477978524	8
W12		4810,05375	982,352876195536	8	2516,81	1023,52933607201	8	3761,71125	789,093664609459	8

Table S11. 2way ANOVA of Mean Velocity (Center-point)

Tukey's multiple comparisons test	Mean diff,	95,00% CI of diff,	Below threshold?	Summary	Adjusted P Value
Before STZ					
Control (DM-) vs. Diabetes mellitus (DM+)	-0,3910	-2,241 to 1,459	No	ns	0,8441
Control (DM-) vs. Treatment (DM+DAPA)	-0,6191	-2,362 to 1,124	No	ns	0,6166
Diabetes mellitus (DM+) vs. Treatment (DM+DAPA)	-0,2282	-1,617 to 1,161	No	ns	0,9024
W4					
Control (DM-) vs. Diabetes mellitus (DM+)	1,790	0,6834 to 2,897	Yes	**	0,0023
Control (DM-) vs. Treatment (DM+DAPA)	0,9772	-0,6919 to 2,646	No	ns	0,2942
Diabetes mellitus (DM+) vs. Treatment (DM+DAPA)	-0,8127	-2,472 to 0,8461	No	ns	0,4108
W8					
Control (DM-) vs. Diabetes mellitus (DM+)	2,533	1,289 to 3,776	Yes	***	0,0003
Control (DM-) vs. Treatment (DM+DAPA)	0,9115	-0,7645 to 2,587	No	ns	0,3498
Diabetes mellitus (DM+) vs. Treatment (DM+DAPA)	-1,621	-3,243 to 0,0004769	No	ns	0,0501
W12					
Control (DM-) vs. Diabetes mellitus (DM+)	3,020	1,772 to 4,268	Yes	****	<0,0001
Control (DM-) vs. Treatment (DM+DAPA)	1,574	0,2468 to 2,901	Yes	*	0,0203
Diabetes mellitus (DM+) vs. Treatment (DM+DAPA)	-1,446	-2,873 to -0,01941	Yes	*	0,0468

Table S12. Row statistics of Mean Velocity (Center-point)

		Control (DM-)			Diabetes mellitus (DM+)			Treatment (DM+DAPA)		
Before STZ		6,6227075	1,58834026063822	8	7,013695	1,18333743815653	8	7,24185625	0,9040856334273	
W4		7,20394875	0,862984201468329	8	5,414	0,827470370431991	8	6,22672875	1,52214248662335	
W8		6,41695625	1,01749383722862	8	3,88424875	0,871864907186847	8	5,50550625	1,4675850443592	
W12		6,37945625	0,851146933058774	8	3,35924875	1,03821993366995	8	4,80550625	1,13806019405123	

Table S13. 2way ANOVA of Cumulative Duration in Center Zone

Tukey's multiple comparisons test	Mean diff,	95,00% CI of diff,	Below threshold?	Summary	Adjusted P Value
Before STZ					
Control (DM-) vs. Diabetes mellitus (DM+)	13,90	-25,81 to 53,61	No	ns	0,6152
Control (DM-) vs. Treatment (DM+DAPA)	13,83	-26,09 to 53,75	No	ns	0,6236
Diabetes mellitus (DM+) vs. Treatment (DM+DAPA)	-0,07000	-22,77 to 22,63	No	ns	>0,9999
W4					
Control (DM-) vs. Diabetes mellitus (DM+)	14,80	-16,71 to 46,30	No	ns	0,4379
Control (DM-) vs. Treatment (DM+DAPA)	7,505	-13,20 to 28,21	No	ns	0,6196
Diabetes mellitus (DM+) vs. Treatment (DM+DAPA)	-7,290	-39,09 to 24,51	No	ns	0,8129
W8					
Control (DM-) vs. Diabetes mellitus (DM+)	35,03	16,87 to 53,18	Yes	***	0,0005
Control (DM-) vs. Treatment (DM+DAPA)	22,16	4,456 to 39,85	Yes	*	0,0147
Diabetes mellitus (DM+) vs. Treatment (DM+DAPA)	-12,87	-28,20 to 2,457	No	ns	0,1062
W12					
Control (DM-) vs. Diabetes mellitus (DM+)	45,43	21,84 to 69,01	Yes	***	0,0009
Control (DM-) vs. Treatment (DM+DAPA)	29,80	6,529 to 53,07	Yes	*	0,0147
Diabetes mellitus (DM+) vs. Treatment (DM+DAPA)	-15,63	-28,35 to -2,896	Yes	*	0,0163

Table S14. Row statistics of Cumulative Duration in Center Zone

		Control (DM-)			Diabetes mellitus (DM+)			Treatment (DM+DAPA)		
Before STZ		92,255	37,1723088179513	8	78,355	16,8124911046179	8	78,425	17,8434453751831	8
W4		79,73	15,2900453143307	8	64,935	29,0330092923801	8	72,225	16,3246343209974	8
W8		81,035	15,2492650876783	8	46,01	12,1650506428398	8	58,88	11,2227523998604	8
W12		83,035	21,9665349371005	8	37,61	10,504415398162	8	53,235	8,80696964260206	8

Table S15. 2way ANOVA of Cumulative Duration in Periphery Zone

Tukey's multiple comparisons test	Mean diff,	95,00% CI of diff,	Below threshold?	Summary	Adjusted P Value
Before STZ					
Control (DM-) vs. Diabetes mellitus (DM+)	-58,27	-151,9 to 35,40	No	ns	0,2301
Control (DM-) vs. Treatment (DM+DAPA)	-55,88	-149,5 to 37,77	No	ns	0,2527
Diabetes mellitus (DM+) vs. Treatment (DM+DAPA)	2,390	-10,59 to 15,37	No	ns	0,8791
W4					
Control (DM-) vs. Diabetes mellitus (DM+)	-19,96	-82,52 to 42,61	No	ns	0,6862
Control (DM-) vs. Treatment (DM+DAPA)	-11,63	-69,73 to 46,48	No	ns	0,8553
Diabetes mellitus (DM+) vs. Treatment (DM+DAPA)	8,330	-41,20 to 57,86	No	ns	0,8979
W8					
Control (DM-) vs. Diabetes mellitus (DM+)	-119,5	-184,7 to -54,25	Yes	**	0,0011
Control (DM-) vs. Treatment (DM+DAPA)	-51,96	-89,49 to -14,43	Yes	**	0,0091
Diabetes mellitus (DM+) vs. Treatment (DM+DAPA)	67,53	5,797 to 129,3	Yes	*	0,0338
W12					
Control (DM-) vs. Diabetes mellitus (DM+)	-119,5	-187,6 to -51,33	Yes	**	0,0011
Control (DM-) vs. Treatment (DM+DAPA)	-49,43	-104,0 to 5,131	No	ns	0,0746
Diabetes mellitus (DM+) vs. Treatment (DM+DAPA)	70,03	15,37 to 124,7	Yes	*	0,0154

Table S16. Row statistics of Cumulative Duration in Periphery Zone

		Control (DM-)			Diabetes mellitus (DM+)			Treatment (DM+DAPA)		
Before STZ		462,71	89,9194223418151	8	520,975	11,1025132289946	8	518,585	8,37765565571385	8
W4		504,94	52,2958703641392	8	524,895	42,3269610127095	8	516,565	32,021460214764	8
W8		496,285	35,1363413820262	8	615,77	59,0159678150147	8	548,245	15,8707268714628	8
W12		505,065	52,0133518572738	8	624,52	52,106335233142	8	554,495	16,1871192178049	8

Table S17. 2way ANOVA of Cumulative Duration Moving

Tukey's multiple comparisons test	Mean diff,	95,00% CI of diff,	Below threshold?	Summary	Adjusted P Value
Before STZ					
Control (DM-) vs. Diabetes mellitus (DM+)	23,99	-43,33 to 91,30	No	ns	0,6291
Control (DM-) vs. Treatment (DM+DAPA)	14,77	-44,01 to 73,54	No	ns	0,7896
Diabetes mellitus (DM+) vs. Treatment (DM+DAPA)	-9,220	-71,51 to 53,07	No	ns	0,9196
W4					
Control (DM-) vs. Diabetes mellitus (DM+)	46,79	-45,36 to 138,9	No	ns	0,4009
Control (DM-) vs. Treatment (DM+DAPA)	30,77	-44,94 to 106,5	No	ns	0,5489
Diabetes mellitus (DM+) vs. Treatment (DM+DAPA)	-16,03	-103,2 to 71,12	No	ns	0,8778
W8					
Control (DM-) vs. Diabetes mellitus (DM+)	136,3	31,36 to 241,3	Yes	*	0,0128
Control (DM-) vs. Treatment (DM+DAPA)	90,66	30,98 to 150,3	Yes	**	0,0039
Diabetes mellitus (DM+) vs. Treatment (DM+DAPA)	-45,69	-148,3 to 56,97	No	ns	0,4620
W12					
Control (DM-) vs. Diabetes mellitus (DM+)	176,6	113,0 to 240,2	Yes	****	<0,0001
Control (DM-) vs. Treatment (DM+DAPA)	102,4	37,96 to 166,8	Yes	**	0,0028
Diabetes mellitus (DM+) vs. Treatment (DM+DAPA)	-74,18	-146,1 to -2,295	Yes	*	0,0428

Table S18. Row statistics of Cumulative Duration Moving

		Control (DM-)			Diabetes mellitus (DM+)			Treatment (DM+DAPA)		
Before STZ		458,315	49,1558242443646	8	434,33	53,5467486007721	8	443,55	39,7248867659988	8
W4		455,08	62,7493116638399	8	408,29	76,7491639786947	8	424,315	51,9851866263018	8
W8		473,7	50,5866746993994	8	337,36	96,8349972154401	8	383,045	39,2590775672146	8
W12		471,2	40,9875967301608	8	294,635	54,4188473128303	8	368,815	55,4344954492623	8

Table S19. 2way ANOVA of Cumulative Duration Not Moving

Tukey's multiple comparisons test	Mean diff,	95,00% CI of diff,	Below threshold?	Summary	Adjusted P Value
Before STZ					
Control (DM-) vs. Diabetes mellitus (DM+)	19,44	-29,49 to 68,36	No	ns	0,5272
Control (DM-) vs. Treatment (DM+DAPA)	39,69	-27,89 to 107,3	No	ns	0,3028
Diabetes mellitus (DM+) vs. Treatment (DM+DAPA)	20,25	-38,24 to 78,74	No	ns	0,6041
W4					
Control (DM-) vs. Diabetes mellitus (DM+)	-14,42	-75,06 to 46,22	No	ns	0,8091
Control (DM-) vs. Treatment (DM+DAPA)	6,870	-69,29 to 83,03	No	ns	0,9683
Diabetes mellitus (DM+) vs. Treatment (DM+DAPA)	21,29	-58,34 to 100,9	No	ns	0,7637
W8					
Control (DM-) vs. Diabetes mellitus (DM+)	-67,50	-132,0 to -2,971	Yes	*	0,0409
Control (DM-) vs. Treatment (DM+DAPA)	-45,28	-146,1 to 55,50	No	ns	0,4408
Diabetes mellitus (DM+) vs. Treatment (DM+DAPA)	22,22	-85,59 to 130,0	No	ns	0,8480
W12					
Control (DM-) vs. Diabetes mellitus (DM+)	-106,9	-181,7 to -32,03	Yes	**	0,0081
Control (DM-) vs. Treatment (DM+DAPA)	-63,91	-160,4 to 32,58	No	ns	0,2032
Diabetes mellitus (DM+) vs. Treatment (DM+DAPA)	42,97	-65,50 to 151,4	No	ns	0,5631

Table S20. Row statistics of Cumulative Duration Not Moving

		Control (DM-)			Diabetes mellitus (DM+)			Treatment (DM+DAPA)		
Before STZ		188,385	46,5649724271674	8	168,95	15,3923283303265	8	148,7	55,8943574969782	8
W4		163,695	41,5366822390591	8	178,115	50,3365107480218	8	156,825	68,7014108817487	8
W8		155,825	24,4792804516099	8	223,32	60,8978010393526	8	201,105	96,4317195888217	8
W12		143,075	25,8670397003048	8	249,945	70,9741906812409	8	206,98	92,1840246463562	8

Table S21. 2way ANOVA of CMAP amplitude

Tukey's multiple comparisons test	Mean diff,	95,00% CI of diff,	Below threshold?	Summary	Adjusted P Value
Before STZ					
Control (DM-) vs. Diabetes mellitus (DM+)	-0,03875	-1,321 to 1,243	No	ns	0,9965
Control (DM-) vs. Treatment (DM+DAPA)	-0,2125	-1,449 to 1,024	No	ns	0,8943
Diabetes mellitus (DM+) vs. Treatment (DM+DAPA)	-0,1738	-1,330 to 0,9826	No	ns	0,9186
W4					
Control (DM-) vs. Diabetes mellitus (DM+)	1,968	0,1477 to 3,787	Yes	*	0,0349
Control (DM-) vs. Treatment (DM+DAPA)	1,180	-0,1281 to 2,488	No	ns	0,0786
Diabetes mellitus (DM+) vs. Treatment (DM+DAPA)	-0,7875	-2,735 to 1,160	No	ns	0,5467
W8					
Control (DM-) vs. Diabetes mellitus (DM+)	4,223	2,239 to 6,206	Yes	***	0,0008
Control (DM-) vs. Treatment (DM+DAPA)	3,035	1,356 to 4,714	Yes	**	0,0021
Diabetes mellitus (DM+) vs. Treatment (DM+DAPA)	-1,188	-3,496 to 1,121	No	ns	0,3926
W12					
Control (DM-) vs. Diabetes mellitus (DM+)	5,281	4,492 to 6,071	Yes	****	<0,0001
Control (DM-) vs. Treatment (DM+DAPA)	4,319	3,532 to 5,106	Yes	****	<0,0001
Diabetes mellitus (DM+) vs. Treatment (DM+DAPA)	-0,9625	-1,914 to -0,01131	Yes	*	0,0472

Table S22. Row statistics of CMAP amplitude

		Control (DM-)			Diabetes mellitus (DM+)			Treatment (DM+DAPA)		
Before STZ		10,0475	1,03054423901715	8	10,08625	0,923254143930973	8	10,26	0,840187054003028	
W4		10,0125	0,732934415307985	8	8,045	1,7105888710366	8	8,8325	1,17257652811478	
W8		10,76375	0,444037884484132	8	6,54125	1,89982283008555	8	7,72875	1,60402562768982	
W12		11,0375	0,381491434092188	8	5,75625	0,728205965762042	8	6,71875	0,725503519731543	

Table S23. 2way ANOVA of CMAP duration

Tukey's multiple comparisons test	Mean diff,	95,00% CI of diff,	Below threshold?	Summary	Adjusted P Value
Before STZ					
Control (DM-) vs. Diabetes mellitus (DM+)	0,01375	-0,1811 to 0,2086	No	ns	0,9812
Control (DM-) vs. Treatment (DM+DAPA)	-0,03125	-0,1920 to 0,1295	No	ns	0,8673
Diabetes mellitus (DM+) vs. Treatment (DM+DAPA)	-0,04500	-0,2289 to 0,1389	No	ns	0,7957
W4					
Control (DM-) vs. Diabetes mellitus (DM+)	-0,2213	-0,4801 to 0,03760	No	ns	0,0973
Control (DM-) vs. Treatment (DM+DAPA)	-0,1625	-0,3930 to 0,06796	No	ns	0,1885
Diabetes mellitus (DM+) vs. Treatment (DM+DAPA)	0,05875	-0,2247 to 0,3422	No	ns	0,8512
W8					
Control (DM-) vs. Diabetes mellitus (DM+)	-0,6813	-0,8963 to -0,4662	Yes	****	<0,0001
Control (DM-) vs. Treatment (DM+DAPA)	-0,4013	-0,6361 to -0,1664	Yes	**	0,0016
Diabetes mellitus (DM+) vs. Treatment (DM+DAPA)	0,2800	0,03170 to 0,5283	Yes	*	0,0267
W12					
Control (DM-) vs. Diabetes mellitus (DM+)	-0,8138	-1,048 to -0,5797	Yes	****	<0,0001
Control (DM-) vs. Treatment (DM+DAPA)	-0,5600	-0,6981 to -0,4219	Yes	****	<0,0001
Diabetes mellitus (DM+) vs. Treatment (DM+DAPA)	0,2538	0,02103 to 0,4865	Yes	*	0,0333

Table S24. Row statistics of CMAP duration

		Control (DM-)			Diabetes mellitus (DM+)			Treatment (DM+DAPA)		
Before STZ		2,26875	0,133356396814808	8	2,255	0,161775859050195	8	2,3	0,110194633003868	
W4		2,2775	0,14547950272903	8	2,49875	0,231852015610943	8	2,44	0,198925686052427	
W8		2,275	0,150427960926722	8	2,95625	0,17630634864187	8	2,67625	0,201560873470735	
W12		2,33	0,108496214548843	8	3,14375	0,217054140711482	8	2,89	0,102399776785471	

Table S25. 2way ANOVA of Hot tail immersion test

Tukey's multiple comparisons test	Mean diff,	95,00% CI of diff,	Below threshold?	Summary	Adjusted P Value
Before STZ					
Control (DM-) vs. Diabetes mellitus (DM+)	-0,2575	-0,5154 to 0,0003990	No	ns	0,0504
Control (DM-) vs. Treatment (DM+DAPA)	-0,09250	-0,3654 to 0,1804	No	ns	0,6516
Diabetes mellitus (DM+) vs. Treatment (DM+DAPA)	0,1650	-0,03496 to 0,3650	No	ns	0,1126
W4					
Control (DM-) vs. Diabetes mellitus (DM+)	0,8050	0,4024 to 1,208	Yes	**	0,0010
Control (DM-) vs. Treatment (DM+DAPA)	0,5200	0,08790 to 0,9521	Yes	*	0,0190
Diabetes mellitus (DM+) vs. Treatment (DM+DAPA)	-0,2850	-0,5550 to -0,01504	Yes	*	0,0389
W8					
Control (DM-) vs. Diabetes mellitus (DM+)	1,025	0,7111 to 1,339	Yes	****	<0,0001
Control (DM-) vs. Treatment (DM+DAPA)	0,7363	0,4883 to 0,9842	Yes	****	<0,0001
Diabetes mellitus (DM+) vs. Treatment (DM+DAPA)	-0,2888	-0,5612 to -0,01630	Yes	*	0,0381
W12					
Control (DM-) vs. Diabetes mellitus (DM+)	1,489	1,219 to 1,759	Yes	****	<0,0001
Control (DM-) vs. Treatment (DM+DAPA)	1,215	0,9607 to 1,469	Yes	****	<0,0001
Diabetes mellitus (DM+) vs. Treatment (DM+DAPA)	-0,2738	-0,5065 to -0,04105	Yes	*	0,0210

Table S26. Row statistics of Hot tail immersion test

		Control (DM-)			Diabetes mellitus (DM+)			Treatment (DM+DAPA)		
Before STZ		2,66625	0,236397818457423	8	2,92375	0,129497655797867	8	2,75875	0,170749398660309	[illegible]
W4		2,51	0,383592045202787	8	1,705	0,122007025558835	8	1,99	0,251054917156728	[illegible]
W8		2,7025	0,226195238045114	8	1,6775	0,25223288558677	8	1,96625	0,127832200280569	[illegible]
W12		2,75625	0,219215321414488	8	1,2675	0,191963538204525	8	1,54125	0,16110666386856	[illegible]

Table S27. 2way ANOVA of Von Frey Test

Tukey's multiple comparisons test	Mean diff,	95,00% CI of diff,	Below threshold?	Summary	Adjusted P Value
Before STZ					
Control (DM-) vs. Diabetes mellitus (DM+)	0,02500	-0,1263 to 0,1763	No	ns	0,9026
Control (DM-) vs. Treatment (DM+DAPA)	0,01250	-0,1378 to 0,1628	No	ns	0,9742
Diabetes mellitus (DM+) vs. Treatment (DM+DAPA)	-0,01250	-0,1575 to 0,1325	No	ns	0,9724
W4					
Control (DM-) vs. Diabetes mellitus (DM+)	0,5063	0,3417 to 0,6708	Yes	****	<0,0001
Control (DM-) vs. Treatment (DM+DAPA)	0,3250	0,1429 to 0,5071	Yes	**	0,0011
Diabetes mellitus (DM+) vs. Treatment (DM+DAPA)	-0,1813	-0,3749 to 0,01238	No	ns	0,0679
W8					
Control (DM-) vs. Diabetes mellitus (DM+)	0,8500	0,7314 to 0,9686	Yes	****	<0,0001
Control (DM-) vs. Treatment (DM+DAPA)	0,6813	0,5319 to 0,8306	Yes	****	<0,0001
Diabetes mellitus (DM+) vs. Treatment (DM+DAPA)	-0,1688	-0,3058 to -0,03172	Yes	*	0,0169
W12					
Control (DM-) vs. Diabetes mellitus (DM+)	1,050	0,9330 to 1,167	Yes	****	<0,0001
Control (DM-) vs. Treatment (DM+DAPA)	0,8313	0,6596 to 1,003	Yes	****	<0,0001
Diabetes mellitus (DM+) vs. Treatment (DM+DAPA)	-0,2188	-0,3828 to -0,05470	Yes	*	0,0111

Table S28. Row statistics of Von Frey Test

	Control (DM-)			Diabetes mellitus (DM+)			Treatment (DM+DAPA)		
Before STZ	1,23125	0,119335960332883	8	1,20625	0,111603571371427	8	1,21875	0,109991882817389	8
W4	1,18125	0,113192314226718	8	0,675	0,136277028773849	8	0,85625	0,158108235983184	8
W8	1,2875	0,102643627594285	8	0,4375	0,0744023809142845	8	0,60625	0,123743686707646	8
W12	1,225	0,1	8	0,175	0,0755928946018454	8	0,39375	0,152215777293758	8

Table S29. Ordinary one-way ANOVA of Nerve tissue density in the skin

Tukey's multiple comparisons test	Mean diff,	95,00% CI of diff,	Below threshold?	Summary	Adjusted P Value
Control (DM-) vs. Diabetes mellitus (DM+)	0,008368	0,003787 to 0,01295	Yes	***	0,0004
Control (DM-) vs. Treatment (DM+DAPA)	0,003699	-0,0008823 to 0,008280	No	ns	0,1285
Diabetes mellitus (DM+) vs. Treatment (DM+DAPA)	-0,004669	-0,009250 to -8,769e-005	Yes	*	0,0453

Table S30. Row statistics of Nerve tissue density in the skin

	Control (DM-)	Diabetes mellitus (DM+)	Treatment (DM+DAPA)
Number of values	8	8	8
Minimum	0,01830	0,01192	0,01558
25% Percentile	0,01941	0,01257	0,01672
Median	0,02294	0,01522	0,01966
75% Percentile	0,02643	0,01779	0,02337
Maximum	0,03264	0,01930	0,02435
Mean	0,02361	0,01524	0,01991
Std. Deviation	0,004669	0,002685	0,003261
Std. Error of Mean	0,001651	0,0009492	0,001153
Lower 95% CI	0,01971	0,01300	0,01719
Upper 95% CI	0,02751	0,01749	0,02264

Table S31. Ordinary one-way ANOVA of Neuronal oxidative stress

Tukey's multiple comparisons test	Mean diff,	95,00% CI of diff,	Below threshold?	Summary	Adjusted P Value	
Control (DM-) vs. Diabetes mellitus (DM+)	-18,66	-25,17 to -12,14	Yes	****	<0,0001	A-B
Control (DM-) vs. Treatment (DM+DAPA)	-10,40	-16,92 to -3,889	Yes	**	0,0017	A-C
Diabetes mellitus (DM+) vs. Treatment (DM+DAPA)	8,253	1,738 to 14,77	Yes	*	0,0117	B-C

Table S32. Row statistics of Neuronal oxidative stress

	Control (DM-)	Diabetes mellitus (DM+)	Treatment (DM+DAPA)
Number of values	8	8	8
Minimum	4,393	16,41	14,53
25% Percentile	5,133	22,16	15,04
Median	8,846	26,32	18,97
75% Percentile	9,898	31,72	22,70
Maximum	15,41	40,49	24,14
Mean	8,553	27,21	18,96
Std. Deviation	3,518	7,303	3,805
Std. Error of Mean	1,244	2,582	1,345
Lower 95% CI	5,612	21,11	15,78
Upper 95% CI	11,49	33,32	22,14

Table S33. Ordinary one-way ANOVA of Nerve fibrosis

Tukey's multiple comparisons test	Mean diff,	95,00% CI of diff,	Below threshold?	Adjusted P Value

Control (DM-) vs. Diabetes mellitus (DM+)	-0,1583	-0,2375 to -0,07920	Yes	0,0002
Control (DM-) vs. Treatment (DM+DAPA)	-0,05425	-0,1334 to 0,02490	No	0,2186
Diabetes mellitus (DM+) vs. Treatment (DM+DAPA)	0,1041	0,02496 to 0,1832	Yes	0,0088

Table S34. Row statistics of Nerve fibrosis

	Control (DM-)	Diabetes mellitus (DM+)	Treatment (DM+DAPA)
Number of values	8	8	8
Minimum	0,05100	0,1882	0,1019
25% Percentile	0,06900	0,2018	0,1243
Median	0,1150	0,2492	0,1623
75% Percentile	0,1505	0,3677	0,2136
Maximum	0,1750	0,4031	0,2507
Mean	0,1135	0,2718	0,1677
Std. Deviation	0,04407	0,08517	0,05133
Std. Error of Mean	0,01558	0,03011	0,01815
Lower 95% CI	0,07666	0,2006	0,1248
Upper 95% CI	0,1503	0,3430	0,2107
